# ATM and ATR, two central players of the DNA damage response, are involved in the induction of systemic acquired resistance by extracellular DNA, but not the plant wound response

**DOI:** 10.3389/fimmu.2023.1175786

**Published:** 2023-05-15

**Authors:** Isaac Vega-Muñoz, Alfredo Herrera-Estrella, Octavio Martínez-de la Vega, Martin Heil

**Affiliations:** ^1^ Laboratorio de Ecología de Plantas, Departamento de Ingeniería Genética, Centro de Investigación y de Estudios Avanzados (CINVESTAV)—Unidad Irapuato, Irapuato, GTO, Mexico; ^2^ Laboratorio Nacional de Genómica para la Biodiversidad, Centro de Investigación y de Estudios Avanzados (CINVESTAV)—Unidad de Genómica Avanzada, Irapuato, GTO, Mexico

**Keywords:** damaged-self recognition, defence signalling, DNA-triggered immunity, eDNA, Mazzoleni effect, plant immune response to DNA, innate immunity

## Abstract

**Background:**

The plant immune response to DNA is highly self/nonself-specific. Self-DNA triggered stronger responses by early immune signals such as H_2_O_2_ formation than nonself-DNA from closely related plant species. Plants lack known DNA receptors. Therefore, we aimed to investigate whether a differential sensing of self-versus nonself DNA fragments as damage- versus pathogen-associated molecular patterns (DAMPs/PAMPs) or an activation of the DNA-damage response (DDR) represents the more promising framework to understand this phenomenon.

**Results:**

We treated *Arabidopsis thaliana* Col-0 plants with sonicated self-DNA from other individuals of the same ecotype, nonself-DNA from another *A. thaliana* ecotype, or nonself-DNA from broccoli. We observed a highly self/nonself-DNA-specific induction of H_2_O_2_ formation and of jasmonic acid (JA, the hormone controlling the wound response to chewing herbivores) and salicylic acid (SA, the hormone controlling systemic acquired resistance, SAR, to biotrophic pathogens). Mutant lines lacking Ataxia Telangiectasia Mutated (ATM) or ATM AND RAD3-RELATED (ATR) – the two DDR master kinases – retained the differential induction of JA in response to DNA treatments but completely failed to induce H_2_O_2_ or SA. Moreover, we observed H_2_O_2_ formation in response to *in situ*-damaged self-DNA from plants that had been treated with bleomycin or SA or infected with virulent bacteria *Pseudomonas syringae* pv. tomato DC3000 or pv. glycinea carrying effector avrRpt2, but not to DNA from H_2_O_2_-treated plants or challenged with non-virulent *P. syringae* pv. glycinea lacking avrRpt2.

**Conclusion:**

We conclude that both ATM and ATR are required for the complete activation of the plant immune response to extracellular DNA whereas an as-yet unknown mechanism allows for the self/nonself-differential activation of the JA-dependent wound response.

## Graphical Abstract

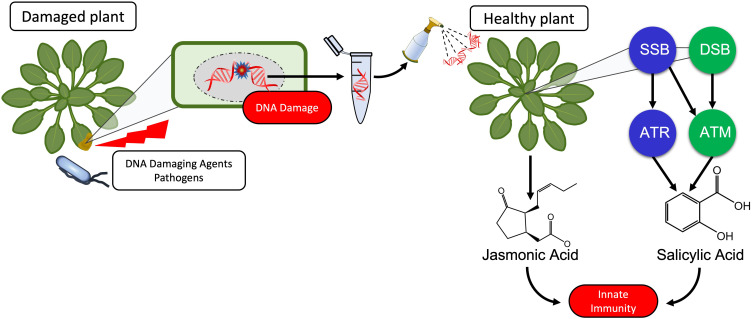

## 1 Introduction

The immunogenic effects of DNA are known since Alick Isaacs’ group reported in 1963 on nonself-nucleic acids as a stimulus to produce interferon (1, 2) although it is worth to note that already Mechnikov in his Nobel Speech in 1908 had mentioned “surgeons who introduce … nucleic acid or other substance, with the object of bringing to the scene a protective army of phagocytes to ward the microbes off” (3). Since then, tremendous progress has been achieved in our understanding of how the mammalian immune system detects bacterial or viral nucleic acids as pathogen-associated molecular patterns (PAMPs) in order to mount an adequate immune response (4–9). “The dark side of DNA” (10) - immune responses to self-DNA – has only recently been considered feasible, violating the immunological paradigm of self-tolerance and thereby sparking controversy. Inappropriate activation of DNA sensors such as cyclic GMP-AMP synthase (cGAS) or Toll-like receptor (TLR 9) by self-DNA has been related to aberrant type I interferon signalling and ongoing inflammation in autoimmune and autoinflammatory disorders as well as in infectious (usually viral) diseases (11–15). Nevertheless, the preference of TLR9 for unmethylated CpG motifs and the expression of cGAS and other DNA sensors in the cytoplasm usually enables the mammalian immune system to limit activation by self-DNA to situations in which delocalized fragments of self-DNA are sensed as a ‘damage-associated molecular patterns’ (DAMPs): endogenous molecules that have an ‘all-day job’ in the intact cell but serve as danger signals when they appear in aberrant compartments (16). A search in Clarivate Web of Science™ (17) for ‘DNA sensing’ currently yields over 25,000 results (search term ‘DNA sensing’ in ‘topic’ performed on 29^th^ of January 2023). By contrast, only a handful of studies over the last 15 years have investigated DNA-activated immunity in plants (**Table 1**).

**Table 1:** Reports on plant immune responses to exogenously applied DNA.

Model	Self-DNA	Nonself-DNA	Response	Result	Conclusion	Ref.
*A. thaliana* Col-0	No	Plasmid and genomic bacterial DNA	H_2_O_2_ formation, callose deposition and inhibition of seedling growth	Enzymatically digested plasmid or bacterial genomic DNA was active, but intact DNA was inactive. Enzymatic methylation of 5’-CG-3’ or cleavage of 5’-CCGG-3’reduced the immunogenic activity	Bacterial linear DNA containing unmethylated CpG motifs has immunogenic properties in plants.	(9)
Several plant species	Yes	Mixture of several plant species	Root and seedling growth inhibition	Self-DNA inhibited root growth in a concentration-dependent manner while the mixture of nonself-DNA did not.	Extracellular DNA has species-specific inhibitory effects on plants.	(18)
*A. thaliana* Col-0	No	*Ps*t DC3000	Disease severity	Bacterial DNA did not trigger a significant reduction of disease severity	Bacterial RNA, but not DNA, triggers a plant immune response	(19)
Lima bean (*P. lunatus*) and maize (*Z. mays*)	Yes	*Spodoptera littoralis* larvae, maize for *P. lunatus*, lima bean for *Z. mays*	Plasma membrane potential depolarization and CA^2+^ fluxes	Perfusion with fragmented self-DNA triggered depolarization and CA^2+^-influxes, no detectable response to nonself-DNA or non-sonicated self-DNA	Fragmentation and the self-origin of DNA are crucial to activate early immune signals.	(20)
Common bean (*P. vulgaris*)	Yes	*P. lunatus* and *Acacia farnesiana*	Seedling growth, H_2_O_2_ formation, MAPK activation, extrafloral nectar (EFN) secretion, resistance to *P. syringae* pv. *syringae*	Self-DNA triggered a dosage-dependent inhibition of seedling growth and induced H_2_O_2,_ MAPKs and resistance to *P. syringae* more strongly than nonself-DNA, and only self-DNA induced EFN secretion	The phylogenetic distance affects the immune response to DNA, in particular in case of the JA-dependent EFN-secretion	(21)
Lettuce (*Lactuca sativa*)	Yes	*Capsicum chinense* and *Acaciella angustissima*	Seedling growth, expression of SOD, CAT and PAL and global level of CpG DNA methylation	Self-DNA and nonself-DNA from *C. chinense* triggered a dosage-dependent inhibition of seedling growth, induced CAT and SOD expression and reduced CpG DNA methylation, while all three types of DNA induced PAL expression	The phylogenetic distance affects the immune response to DNA, but even nonself-DNA from a distant species induces PAL, a SA-synthetic enzyme	(22)
*A. thaliana* Col-0, including mutants mpk3 and npr1-3	No	ssODNs IMT 504 and 2006	Resistance to *B. cinerea* and *Pst* DC3000, stomatal closure, a H_2_O_2_ -dependent response	Both ssODNs induced resistance to *B. cinerea* and *Pst* DC3000 with no detectable difference, and they triggered stomatal closure in a mpk3- and npr1-3 dependent way	DNA triggers resistance to pathogens independently of the presence of CpG motifs but depending on MAPK signalling and NPR1, a central activator of PR-gene expression	(23)
*A. thaliana*	Yes	*Clupea harengus* (fish)	Transcriptomic response, microscopy for DNA uptake.	Nonself-DNA enters root cells and triggers differential expression of ca 6000 genes while self-DNA remains in the apoplast and affects the expression of ca 1500 genes	An as-yet unknown self/nonself-specific transport of DNA that generates differential ## that might contribute to the differential immune responses	(24)
Tomato (*S. lycopersicum*)	Yes	No	Membrane depolarization, CA^2+^ fluxes, H_2_O_2_ formation, and RNAseq of transcriptomic responses	Self-DNA triggered membrane depolarization, CA^2+^-influxes, increased H_2_O_2_ levels and induced enhanced expression of genes related to plant defence and phytohormones, but reduced expression of shock proteins, heat shock factors, pumps and photosynthesis-related genes	The physiological and transcriptomic changes in response to self-DNA treatment are consistent with general patterns of induced plant resistance	(25)
*A. thaliana* Col-0	Yes	Various *A. thaliana* ecotypes and other plant species:	H_2_O_2_ formation. expression of MPK3, OXI1 and CML37, resistance to *B. cinerea*, *Hyaloperonospora arabidopsidis, Myzus persicae and Pst* DC3000,	Self-DNA induced H_2_O_2_, resistance to biological enemies and marker gene expression, nonself-DNA from other ecotypes had similar effects, while nonself-DNA from other species had lower or no effects on marker gene expression	The phylogenetic distance affects the immune response to DNA	(26)
*Capsicum annuum*	No	*Phytophthora capsici* *F. oxysporum* *Rhizoctonia solani*	Phenolics and total flavonoids contents, PAL, Mn-SOD and CHS expression, disease resistance	Pathogen eDNA treatment reduce severity of disease and plant mortality, while also increasing levels of total phenols and flavonoids, and expression of PAL and CHS	Using pathogen eDNA as treatment induced the plant immune system, reducing mortality and disease severity.	(27)
*Musa acuminata*	No	*F. oxysporum*	Disease resistance, ROS detection, PR1, chitinase 1, SOD, CAT gene expression	High concentrations of pathogen DNA increased plantlets’ disease resistance. ROS accumulation (24 h) and defence-related genes were highly induced in plantlets after treatment (9 days)	eDNA exhibits antifungal activity combining an inhibition of fungal growth with a priming of the plant immune system.	(28)
*Capsicum annuum*	No	*Phytophtora capsici*	Resistance to *P. capsici*, increment in phenols and total flavonoids	Low concentrations of pathogen eDNA (2 µg ml^-1^) induced resistance against the pathogen while higher concentrations (60 and 100 µg ml^-1^), on the contrary, made the plant more susceptible	The dose versus quantity of inoculum may interfere with the plant-pathogen interaction.	(29)
*Alnus glutinosa*	Yes	*Festuca drymeja*	Root growth inhibition in open and closed systems	Self-DNA application affected the root health only in closed systems.	Self-DNA but not nonself-DNA, caused toxic effects on the roots in closed systems while in open systems the harmful effects of self-DNA were dramatically reduced.	(30)
*Chlamydomonas reinhardtii* *Nannochloropsis gaditana*	Yes	*Sardina pilchardus* (fish)	Culture cell density inhibition, Morphological changes.	Presence of self-DNA affects cell density and generation time in both freshwater and marine microalgae in a concentration-dependent manner as low concentrations (3 µg ml^-1^) favored growth while higher concentrations (10 µg ml^-1^) inhibited it.	Inhibitory effects are dosage-dependent.	(31)
*A. thaliana*	Yes	*Z. mays*, *Clupea harengus* (fish)	Metabolite profiling	Self-DNA treatment induced the four ribonucleosides and its corresponding bases along with AMP, GMP and N6-methyl-AMP.	Self-DNA treatment induces the accumulation of RNA building blocks.	(32)
*S. lycopersicum*	Yes	*F. oxysporum*	ROS formation, CAT, SOD and PAL activity and total phenolics and flavonoids content.	Self-DNA induced the phenylpropanoid pathway, nonself-DNA induced an intense response of the antioxidant system and lower ROS formation.	The intensity of the immune responses depends on the plant ontogenetic stage. Adult plants have a higher response threshold, thus the stimulus must be higher to obtain the same results for younger plants.	(33)
Peach fruits (*Prunus persica*)	Yes	No	Resistance to *Rhizopus stolonifera*, RNAseq of transcriptomic responses	Self-DNA treatments of peach fruits enhanced resistance to fungal infection *via* a pathway that depends on the co-receptor BAK1 and includes ethylene-dependent genes	Self-DNA can be used for postharvest biocontrol of fungal infections, resistance is linked to ethylene signalling	(34)
*A. thaliana* Col-0 and *S. lycopersicum*, incl. mutants affected in JA synthesis or signalling	Yes	Different ecotypes/cultivars, different species from same genus/family, species from different families	Root growth inhibition, ROS formation, gene expression, resistance to *B. cinerea*	Self-DNA triggered a dosage-dependent inhibition of root growth in seedlings, increased levels of H_2_O_2_, of a JA precursor, of JA-Ile and of the expression of JA-related genes. Lower/no growth inhibition in response to nonself-DNA from related/distant species and in JA-signalling mutants treated with self-DNA.	Confirms that the dosage and the taxonomical distance affect the immune response to DNA and indicates that the JA-controlled wound response is involved in this response.	(35)
*P. vulgaris*	Yes	*P. lunatus* and *Acacia farnesiana, as well as self-DNA with enzymatically manipulated CpG methylation*	Induction of ROS, JA and SA, resistance to 1 herbivore, 4 fungi and 4 bacteria, seed production in the open field	Self/nonself-specific induction of JA and herbivore resistance and enhancement of seed yield but non-specific induction of SA and resistance to microbial pathogens by all three types of DNA tested. Self-DNA with enzymatically altered CpG methylation had lower but still significant ROS-inducing effects	The presence of unmethylated CpG motifs contributes to the immunogenic effects of DNA. DNA triggers a self/non-self-specific induction of the WR and a non-specific induction of SAR, leading to net effects that can be adaptive under natural enemy pressure.	(36)

A, *Arabidopsis*; AMP, Adenosine monophosphate; B., *Botrytis*; CAT, catalase; CML37, calmodulin like 37; CHS, chalcone synthase; F., *Fusarium*; GMP, guanosine monophosphate; JA, jasmonic acid; Mn-SOD, Manganese superoxide dismutase; MPK3, Mitogen-activated protein kinase 3; NPR1, no-pr-proteins 1; OXI-1, oxidative signal-inducible 1; PAL, phenylalanine ammonia lyase; *P. lunatus/P.vulgaris, Phaseolus lunatus/P.vulgaris*; PR, pathogenesis-related; Pst, *Pseudomonas syringae* pathovar tomato; S., *Solanum*; SA, salicylic acid; SOD, superoxide dismutase; Z., *Zea*.

Treatments with plant DNA, genomic or plasmid bacterial DNA, or synthetic single stranded oligodeoxynucleotides (ssODNs), were shown to trigger early immune responses that are highly conserved between plants and mammals, including Ca^2+^ fluxes, membrane depolarization, the formation of reactive oxygen species (ROS) such as hydrogen peroxide (H_2_O_2_), or the activation of mitogen-activated protein kinases (MAPKs) (9, 20–22, 25, 27, 34, 35). The responses include massive transcriptomic reprogramming (24, 25, 35), and studies at the phenotypic level reported not only increased immunity (‘resistance’) to microbial pathogens, but also increased ‘defence’ to plant-specific pests such as leaf and sap-sucking herbivores (21, 23, 26, 27, 29, 33–36). Strikingly, stronger responses to self- in comparison to nonself-DNA were reported in most, although not all, of the studies that compared DNA from different sources. Even the ‘Mazzoleni-effect’ – a dosage-dependent inhibition of growth by self-DNA, but not nonself-DNA that has been described originally by the group of Stefano Mazzoleni – could subsequently be confirmed in different models, including a tree (*Alnus glutinosa*), freshwater and marine algae and the nematode, *Caenorhabditis elegans* (18, 21, 22, 30, 31, 35, 37, 38). Differential responses to self- versus nonself-DNA are significant even when nonself-DNA from closely related genotypes (species of the same genus or ecotypes of the same species) is used (21, 26, 31, 35, 36, 38).

Plants coordinate their responses to attack by biological enemies *via* two major signalling pathways: the jasmonic acid (JA)-dependent wound response (WR) and salicylic acid (SA)-dependent systemic acquired resistance (SAR). The WR is activated upon mechanical damage, feeding by herbivores or infection by necrotrophic pathogens that cause intensive tissue damage. Detection of these events depends on membrane-bound pattern recognition receptors (PRRs) that sense molecular nonself patterns from the herbivore (i.e., herbivore-associated molecular patterns, HAMPs) or DAMPs that are released upon cell disruption, including sucrose, glutamate, cell wall components such as oligogalacturonides, signalling peptides, extracellular ATP, high mobility group box proteins (HMGB) and – most likely - self-DNA (21, 39–44). Upon sensing of HAMPs or DAMPs, metabolic reprogramming allows for the induction of numerous JA-responsive mechanical, biochemical and chemical mechanisms that exert repellent, toxic or otherwise detrimental effects on enemies whose feeding habit exposes them to the intracellular contents of their host. In contrast, SAR is activated in response to biotrophic pathogens and sucking herbivores that generate only minor physical damage. Perception of these events also relies on PRR sensing of PAMPs and the subsequent sensing of effectors which lead to accumulation of ROS, phytoalexins, programmed cell death and the systemic induction of multiple SA-dependent pathogenesis-related (PR) proteins including PR1, chitinases, β-1,3-glucanases and thaumatin-like proteins. Both the WR and SAR usually lead to a systemic so-called ‘pattern-triggered immunity’ (PTI) even after local attack. Considering the different roles of DAMPs and PAMPs in the activation of PTI, it seems reasonable to assume that self- and nonself DNA is sensed as a DAMP or a PAMP respectively, similar to the roles that place DNA sensors such as cGAS and TLR9 inflammasome-forming DNA receptors at the centre of mammalian innate immunity to pathogens, cancer and damage by abiotic factors (6, 15, 45, 46).

Alternatively, exogenously applied DNA could trigger the DNA damage response (DDR): A highly conserved system enabling eukaryotes to detect and repair damage to their genomic, plastid and mitochondrial DNA resulting from the effects of external abiotic and biotic factors but also during vital processes including photosynthesis, oxidative phosphorylation and DNA transcription and synthesis (47–51). Pharmacological studies that link DNA damage to the resistance of pea plants to fungal pathogens date back to the early 1970s (52–54), reviewed in (55, 56). Since then, it has become increasingly evident that activation of the DDR and of innate immunity are closely related (57–62). Current knowledge does not provide sufficient evidence to favour one of the proposed models (DAMP/PAMP-triggered PTI versus DDR activation) over the other. In principle, PTI, being a mechanism which evolved for the perception of exogenous threats, could be considered more likely to respond in a self/nonself-specific way to exogenously applied DNA fragments than the DDR, which evolved to detect damaged self-DNA. However, on the one hand, an induction by DNA has been reported for traits that are typically controlled *via* WR as well as SAR. Further, more recent support for the PTI model comes from a study that linked resistance-induction by self-DNA in peach (*Prunus persica*) fruits to the brassinosteroid insensitive 1-associated receptor kinase 1 (BAK1) – a cytoplasmatic co-receptor that interacts with various PRRs of DAMPs and PAMPs (34) - and from two studies that reported self-DNA-induced JA-synthesis in *A. thaliana* and common bean (*Phaseolus vulgaris*) (35, 36). On the other hand, self-/nonself specific effects are mainly reported for early, not enemy-specific immune signals which have also been reported for the DDR, and as far as we know, no PRRs that could act as dsDNA receptors have been reported for plants or could be predicted by *in silico* approaches (4, 63–65). Therefore, the main goal of our present study was to investigate which of the before mentioned models provides the more promising framework to guide future research and to compare the effects of sonicated DNA to DNA fragments produced under more realistic scenarios. We hypothesized that the DDR, as the more conserved mechanism of sensing damaged DNA, should play a role in the innate immunity induction by fragmented DNA but perhaps not be sufficient to control the differential responses to DNA from different sources.

To characterise the self/nonself-specific immune response to DNA in the model plant, *Arabidopsis thaliana* Col-0, we treated Col-0 plants with sonicated self-DNA from other plants of the same ecotype, nonself-DNA from *A. thaliana* ecotype Cape Verde islands (Cvi-0) or nonself-DNA from broccoli (*Brassica oleracea* var. italica, ‘Br’) and quantified H_2_O_2_ as an early, nonspecific signal and the hormones, JA and SA, as later signals specifically involved in the WR or SAR, respectively. ROS including H_2_O_2_ are known as endogenous DNA-damaging factors that are generated during all basal metabolic processes that include oxidative phosphorylation, but they also serve as local defence against invading pathogens and play a central role as signals that trigger the local and in the form of the ROS-wave, systemic activation of innate immune responses (66, 67). Although ROS had originally been considered mainly in the context of SAR, more recent evidence demonstrates their role in WR. The cognate receptor of eATP, a well-established DAMP in plants, is a lectin-type receptor like kinase that upon sensing eATP activates the ROS-wave by inducing NADPH oxidase-dependent superoxide activation (68). The role of JA as the mobile signal in the WR and the essential role of SA in the systemic tissue response (although not as the mobile signal) are generally accepted. SA is important for SAR activation since the SA receptor, “NO pathogenesis-related protein 1 (NPR1)” serves as a transcription factor for PR1 expression (69, 70). In homeostasis, NPR1 is a cytoplasmic oligomer, but upon binding SA, NPR1 monomers are formed and translocated to the nucleus, where they bind to the PR1 promoter facilitating its expression (69). NPR1 also seems to be involved in coordinating the WR-SAR trade-off. However, doubts remain concerning to which precursors liberated from different substrates and two major biosynthetic pathways contribute to the accumulation of JA and SA in response to wounding and infection, respectively (71–78). Therefore, we opted for a quantification of the hormones themselves.

As a first step to elucidate a potential role for the DDR, we used T- DNA insertion lines of the two master kinases: Ataxia Telangiectasia Mutated (ATM) and ATM AND RAD3-RELATED (ATR) (47, 51, 62, 79, 80). Although neither of these kinases are directly involved in damage recognition, they associate with DNA binding proteins, recruit and/or activate additional proteins and thus, organise large protein complexes that are required for a rapid and efficient repair. While ATR is mainly activated by single-strand breaks (SSBs), ATM is activated by double-strand breaks (DSBs) (47, 51, 62, 79, 80).

In WT, JA increased in response to DNA from all three sources, although with self/nonself-specific quantitative differences, whereas SA and H_2_O_2_ increased only in response to DNA from the two *A. thaliana* ecotypes. Surprisingly, the *atm* and *atr* mutants were unaffected in the self/nonself-specific induction of JA, whereas both failed to respond to DNA with a detectable increase in H_2_O_2_ or SA. Interestingly, all three sources of DNA induced phenotypic resistance to *Pst* DC3000 in *atr* but not *atm* mutant lines. We interpret these results as a preliminary demonstration of a role for the DDR in the induction of SA by exogenous DNA, whereas a DDR-independent mechanism enables plants to perceive self-DNA as a DAMP to activate a JA-dependent wound response.

## 2 Materials and methods

### 2.1 Plant material and bacterial strains

Wild type *Arabidopsis thaliana* Col-0 plants were donated by Stefano Mazzoleni (Naples, Italy), T-DNA insertion lines *atm-1* (SALK_040423C) and *atr-2* (SALK_032841C) were acquired from European *Arabidopsis* Stock Centre, Nottingham, UK (NASC), *A. thaliana* ecotype Cape Verde islands (Cvi-0) were donated by Jean-Philippe Vielle-Calzada (Irapuato, Mexico) and *Brassica oleracea* seeds were purchased from Rancho Los Molinos (Tepoztlán, Morelos, Mexico). We used *A. thaliana* Col-0 as sources of self -DNA and *A. thaliana* Cvi-0 and *B. oleracea*as sources of non-self-DNA. *A. thaliana* plants were grown in a growth chamber with a photoperiod of 16 h/8 h light/dark at 23°C with 70% relative humidity for 3 week in a substrate composed of 3:1:1 (v:v:v) of Sunshine Mix #3 (Sungro horticulture Agawam, MA, USA), vermiculite (Sungro) and perlite (Termolita, Santa Catarina, Mx). *B. oleracea* plants were cultivated in the same substrate in a greenhouse under natural light and photoperiod conditions at an average temperature of 28°C (day) and 20°C (night). T-DNA insertion lines were genotyped using the protocol recommended by NASC (81) and the primers listed in **Supplementary Figure S5**. PCR conditions using a C1000 Thermal cycler (Biorad, Hercules, CA, USA) were as follows: initial denaturalization at 94°C for 3 min, followed by 30 cycles as follows: 94°C for 30 s, 55°C (for *atr* samples) or 58°C (for *atm* samples) for 30 s, 72°C for 2 min and a final extension at 72°C for 10 min.

*Pseudomonas syringae* pv. tomato DC3000 (*Pst* DC3000) were donated by John Délano-Frier (Irapuato, Mexico) and *Pseudomonas syringae* pv. glycinea carrying vector pVSP61 [*Psg* (–)] or the vector pV288 containing the effector avrRpt2 [*Psg* (+)] were donated by Andrew F. Bent (Madison, WI, USA) (82). These strains were cultured at 28°C with peptone-yeast-glycerol agar (NYGA) (83) containing 25 µg ml^-1^ kanamycin (Sigma-Aldrich, St. Louis, MO, USA).

### 2.2 Extraction and preparation of DNA

The extraction of DNA from leaves of *A. thaliana* Col-0, *A. thaliana* Cvi-0 and *B. oleracea* was based on a method reported by Healey & col (84). A total of 20 ml of cetyltrimethylammonium bromide (CTAB) buffer (1.5% CTAB, 100 mM Tris-HCl pH 8, 20 mM EDTA pH 8, 1.4 M NaCl and 2% β-mercaptoethanol in water, all from Sigma-Aldrich) were added per 5 g of ground tissue in 50 ml tubes, shaken on a vortex and heated to 65°C for 30 min in a water bath. Then, 20 ml of 24:1 chloroform:isoamyl alcohol (Karal, Leon, Gto, Mexico) was added and shaken on a vortex. The tubes were centrifuged at 3000 g for 5 min at 4°C and the supernatant was transferred to a new 50 ml tube. Next, 20 ml of precooled isopropanol and 2 ml of ammonium acetate 7.5 M (Karal, Leon, Gto, Mexico) were added to the supernatant, which was kept at –20°C. After 1 h, the tubes were centrifuged at 3000 g for 20 min at 4°C, the supernatant was discarded, and the pellet was dried for 5 min at room temperature before washing by adding 5 ml of 70% ethanol (Karal, Leon, Gto, Mexico) and shaking. After further centrifugation at 3000 g for 10 min at 4°C, the supernatant was discarded, the pellet was dried for 5 min and re-suspended in 1 ml of sterile distilled water for subsequent purification using a Maxi DNA purification Kit (Qiagen, Hilden, Germany). The DNA was quantified using a NanoDrop 2000 spectrometer (Thermo Fisher Scientific, Waltham, MA, USA). To obtain fragments shorter than 1000 bp, a solution of 500 μg ml^–1^ of DNA in sterile distilled water was sonicated using a CP505 ultrasonic processor (Cole Parmer, Vernon Hills, IL, USA) for 3:30 min at 55% of amplitude and using pulse mode (1 s pulse ‘On’ and a 1 s pulse ‘Off’). The successful fragmentation of DNA was verified by gel electrophoresis on a 2.2% agarose gel using ethidium bromide staining (Thermo Fisher) (**Supplementary Figure S1**).

### 2.3 Quantification of responding signals

#### 2.3.1 *ROS* (H2O2) levels

To characterise the ROS response to exogenous DNA fragments, two initial experiments were carried out. In the first one, we treated plants with fragmented self-DNA at 5 µg ml^−1^ or 50 µg ml^− 1^ in 0.05% v v^−1^ Tween 20 and sampled at 0, 5, 10, 15, 30, 45 and 60 min after treatment, in the second one, we treated plants with 0, 0.005, 0.05, 0.5, 5 or 50 µg ml^−1^ self-DNA and sampled at 15 min after treatment. The rosettes of 15 plants were cut off at the base, pooled in groups of three rosettes to give 5 biologically independent replicates per time point and ground in liquid nitrogen. Subsequently, we added 0.1% trichloroacetic acid (250 µl) (Sigma-Aldrich), 250 µl of potassium phosphate buffer 10mM, pH 5.8 and 500 µl of sodium iodide 1M (both from JT Baker, Phillipsburg, NJ, USA) to 150 mg of ground tissue, samples were shaken and incubated at 4°C for 10 min in the dark. After centrifugation at 12,000 g for 15 min at 4°C, 200 µl of supernatant were placed in microplate wells and incubated in the dark for 20 min at room temperature. Hydrogen peroxide quantification (85) was carried out using NaI (JT Baker) instead of KI and absorbance was measured at 350 nm in a µQuant microplate reader (BioTek Instruments, Winooski, VT, USA). Samples and blanks were compared to a calibration curve obtained using H_2_O_2_ at concentrations of 0–250 nmol ml^-1^. To visually detect the accumulation of ROS, after 15 min, leaves were stained with 3,3-diaminobenzidine (Sigma-Aldrich) as described previously (86). For subsequent experiments DNA was applied at 5 µg ml^−1^ self-DNA and samples were taken at 15 min after treatment.

#### 2.3.2 Quantification of JA and SA

To quantify the levels of JA and SA, fragmented self and nonself-DNA (5 µg ml^−1^ in 0.05% v v^−1^ Tween 20, Sigma-Aldrich) was sprayed on both sides of the rosette leaves of each plant until the leaf surface was soaked. Samples for JA analysis were collected at 0, 5, 10, 15, 30, 45 or 60 min after treatment and those for SA analysis at 0, 12, 24 or 48 h as described above to yield 5 biologically independent replicates per time point.

The extraction of JA was carried out as described in (87). 0.5 ml of ethyl acetate and 20 µl of 0.1 mg ml^-1^ (9,10- H2)-dihydrojasmonic acid as an internal standard (both from Sigma-Aldrich) were added to 250 mg of tissue, shaken and kept at 4°C overnight. After centrifugation at 14000 g for 15 min at 4°C, the supernatant was collected, and the pellet was re-extracted with 0.5 ml of ethyl acetate and centrifuged. The supernatants were combined and evaporated in a miVac concentrator (GeneVac, Warminster, PA, USA) with gaseous nitrogen. The residue was derivatized by adding 100 µL of N´N´-disopropylethylamine, 100 µl of chloroform and 10 µl of pentafluorobenzyl bromide (Sigma-Aldrich) at 60°C under an extraction hood (88). After 30 min, the resultant liquid was cooled on ice and evaporated with gaseous nitrogen. The residue was re-suspended with 100 µl of HPLC grade methanol (Sigma-Aldrich) and used for JA quantification.

The extraction of SA was based on published methods (89, 90). In brief, 750 µl of 90% methanol and 250 ng ml^-1^ of ortho-anisic acid (as internal standard, Thermo Fisher) were added to 250 mg of ground tissue and extracted at 4°C overnight. After centrifugation at 13000 g for 15 min at 4°C, the supernatant was collected, the pellet was re-extracted with 750 μl of 90% methanol, and both supernatants were combined and evaporated in a miVac Concentrator plus (GeneVac) for 4 h. The residue was re-suspended with 500 μl of trichloroacetic acid 5% and centrifuged at 4000 g for 10 min at 4°C. The supernatant was mixed with two volumes of ethyl acetate-hexane (1: 1 v v-1, Sigma-Aldrich) and incubated at room temperature for 10 min. The organic phase was recovered and dried with gaseous nitrogen. The residue was derivatized by mixing with 20 µl of pyridine (JT Baker) and 80 μl of N,O-bis(trimethylsilyl)trifluoroacetamide (BSTFA; Sigma-Aldrich) and incubated at 80°C for 1 h, using an extraction hood. The resulting mixture was used for SA quantification using gas chromatography.

Both hormones were quantified using gas chromatography - electronic impact ionization mass spectrometry (GC-EIMS) in a 7890A Gas Chromatograph equipped with a DB-1MS UI column (60 m × 0.25 mm × 0.25 µm) coupled to a MSD 5973 mass spectrometer (Agilent Technologies, Santa Clara, CA, USA) in SIM mode, using ions 141, 181, 390 and 392 m z^–1^ for JA and 73, 135, 267 and 282 m z^–1^ for SA. An injection volume of 1 µl of sample was used in the splitless mode. For JA, the operating conditions (91) were an injector temperature of 200°C and an initial oven temperature of 150°C for 3 min, which was then ramped at 4°C min^–1^ to 300°C, with the final temperature maintained for 20 min. For SA we used an injector temperature of 200°C and an initial oven temperature of 150°C for 3 min, which was then ramped at 4°C min^–1^ to 260°C, with the final temperature maintained for 25 min. Helium was used as a carrier gas with a constant flow of 1 ml min^–1^ and standard curves were prepared using pure compounds (JA, Sigma-Aldrich; SA, JT Baker) to quantify the respective amounts of JA and SA based on peak areas with reference to the internal standard.

### 2.4 *In situ* DNA fragmentation by pathogens and DNA-damaging agents

#### 2.4.1 Generation and elution of *in situ* damaged DNA

To elucidate whether the effects of sonicated DNA are similar to those of naturally damaged DNA, we inoculated Col-0 plants with one of three bacterial strains: virulent *Pst* DC3000 and *Psg* (+) or the non-virulent *Psg* (–) (82, 92). The two virulent bacteria, *Pst* DC3000 and *Psg* avrRpt2 + have been reported to inflict damage to the DNA of their host plant (93). For inoculation, bacteria were resuspended in 10 mM MgCl_2_ (Sigma-Aldrich). After adjusting the bacterial suspension following an established protocol (A.F. Bent, personal communication) by quantifying optical density at 600nm (where od 0.1 = bacterial density of 10^8^ cells) in a µQuant microplate reader (BioTek Instruments, Winooski, VT, USA) *A. thaliana* Col-0 plants were inoculated by spraying the rosette with a suspension of 10^8^ colony forming units (CFUs) ml^-1^ in 10 mM MgCl_2_. Alternatively, the plants were syringe infiltrated with 100 µl of an aqueous solution of a DNA-damaging agent (56, 59, 93–95): 0.6 µg ml^-1^ bleomycin (Sigma-Aldrich), 100 µM SA (Sigma-Aldrich), or 80 mM H_2_O_2_ (Jaloma, Guadalajara, Jal, Mexico).

Damaged DNA was retrieved from bacteria-inoculated plants after 7 days and from plants infiltrated with SA, bleomycin and H_2_O_2_ after 24 h, 12 h or 3 h, respectively, adapting a method developed to quantify the leakage of NAD(P) from leaf disks floating on water (96). Thirty leaf discs of 6 mm diameter were harvested from 10 individual plants per treatment and pools of 10 leaf disks were placed in test tubes with 600 µl CTAB buffer. After shaking for 10 minutes, the buffer was recollected in a microcentrifuge tube and the leaked DNA was precipitated with 400 µl of isopropanol and 60 µl of ammonium acetate 7.5 M (Karal Leon, Gto, Mexico) and quantified using a NanoDrop 2000 spectrometer (Thermo Fisher Scientific, Waltham, MA, USA). To control for any artefacts resulting from DNA fragmentation during this process, we applied a mechanical stress control. For this we collected leaf discs from untreated plants, placed them in the buffer and gently pressed them repeatedly between a plastic stick and the tube, avoiding visible tissue disruption.

#### 2.4.2 Analysis of fragmentation and H_2_O_2_-inducing properties of eluted DNA

To evaluate the *in situ* fragmentation caused by the beforementioned treatments in comparison with the pattern observed in sonicated DNA, the DNA was visualized on a 2.2% agarose gel using ethidium bromide staining and the signal intensity of the fragments that fell within a size range of < 1000 bp in each lane was estimated using Image Lab (Biorad). To test these fragments for their ROS-inducing activities, we treated Col-0 plants with the eluted DNA and sampled these plants 15 min later to quantify H_2_O_2_-levels as described in section 2.3.1.

#### 2.4.3 Estimation of total DNA amount

The expected total amount of DNA was calculated based on assuming a total number of 130,000 cells in a leaf with an area of 200 mm^2^ (97) and a 2C-value of 0.32pg (98). Based on these assumptions, we calculated an area of 0.0015 mm^2^ and a radius of 0.022 mm for the average cell, which translates to a total number of 18,400 cells in the entire leaf disc and of 850 cells in the circumference, with an expected total amount of 5.88 ng DNA in the entire disc and of 0.27 ng in the circumference (**Table 2**).

**Table 2:** Parameters and calculated values to estimate the expected total amount of DNA in leaf discs of 6mm.

Parameter	Value	Unit
leaf area	200	mm^2^
total cells	130000	cells
cell area	0.001538462	mm^2^
cell radius	0.022129336	mm
2C-value	0.32	pg
total DNA in leaf	41600	pg
total DNA in leaf	41.6	ng
leaf circle radius	3	mm
leaf circle area	28.27433388	mm^2^
cells by leaf circle	18378.31702	cells
total DNA in leaf circle	5881.061448	pg
total DNA in leaf circle	5.881061448	ng
circle circunference	18.84955592	mm
cells in circunference	851.7903934	cells
DNA in circunference	272.5729259	pg
DNA in circunference	0.272572926	ng

### 2.5 Testing for TLR9-like responses

#### 2.5.1 Immunostimulatory oligonucleotides

To determine importance of CpG motifs (23), the ODNs IMT504 (5’-CATCATTTTGTCATTTTGTCATT-3’) and 2006 (5’- TCGTCGTTTTGTCGTTTTGT-3’) and their complementary sequences (IMT504c: 5’- AATGACAAAATGACAAAATGATG-3’; 2006c: 5’-ACAAAACGACAAAACGACGA-3’) were purchased from Sigma Aldrich and used as single stranded DNA (ssDNA). To generate double stranded DNA (dsDNA), each ODN was annealed with its complementary sequence in a C1000 Thermal cycler (Biorad) by heating to 95°C for 3 minutes and cooling to room temperature.

#### 2.5.2 Generation of CpG-methylated DNA

In order to obtain DNA with completely methylated CpG motifs, sonicated DNA of Col-0, Cvi-0 or Br was used as substrate for the CpG DNA methyltransferase from *Spiroplasma* sp. strain MW1 (M.SssI, Thermo Scientific #EM0821) (99). Methylation was performed according to the product manual. To evaluate the degree of methylation, non-fragmented DNA was treated with M.SssI and aliquots of 1 µg µl^–1^ of the product were digested with each of two restriction enzymes, MspI (Thermo-Scientific #ER0541) and HpaII (Invitrogen INVN093-6), according to the product manuals (**Supplementary Figure S2**).

#### 2.5.3 Generation of unmethylated DNA

To generate unmethylated DNA, genome amplification was performed using 15-mer random primers and non-fragmented DNA of Col-0, Cvi-0 or Br as a template for amplification *via* PCR in a C1000 Thermal cycler (Biorad), using the following conditions: initial denaturalization at 94°C for 2 min followed by 50 cycles as follows: 94°C for 1 min, alignment ramping from 28 to 55°C (0.1 °C s^-1^), 55°C for 4 min, extension at 68°C for 30 s and a final extension at 68°C for 8 min. PCR products were separated by gel electrophoresis on a 2.2% agarose gel.

### 2.6 Statistical analyses

All statistical analyses and plots were performed using R version 4.2.0 (100) with Rstudio version 2022.12.0.353 (101) as integrated development environment. Global data from each experimental set was tested by either one- or two-way analysis of variance (ANOVA), when significant differences were found (p < 0.05) a Tukey test was performed. For the dose response curve analyses, we used the drc package (102), while the tidyverse (103), multcompView (104), ggbeeswarm (105), ggtext (106), ggrepel (107) and rstatix (108) packages were used for creating the plots and general data wrangling. R scripts used for the statistical analyses and for creating the figures are available at GitHub as IsaacVegaM/PAPER_Vega_2023_exDNA_DDR.

## 3 Results

### 3.1 The ROS response to DNA is time- and dosage-dependent

**Figure 1:**
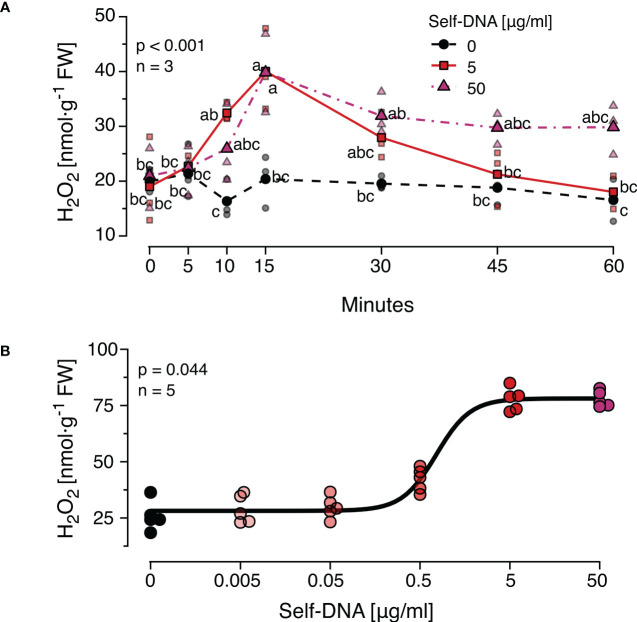
The *Arabidopsis thaliana* ROS response to exogenous DNA. The level of ROS in [nmol H_2_O_2_ g^-1^ leaf fresh weight] was determined at different time points **(A)** or 15 min **(B)** after treating *A*. *thaliana* Col-0 plants with a solution of self-DNA in 0.05% v v^−1^ Tween 20. Controls (black symbols) were treated with 0.05% v v^−1^ Tween 20 in water. Different DNA concentrations are indicated as different saturations of red, symbols represent individual data points. In **(A)** dotted lines represent means and different letters indicate statistically significant differences at each time point among plants treated with different concentrations (p < 0.05, *post hoc* Tukey tests; p < 0.001 for time and for concentration, and p = 0.002 for the interaction according to two-way ANOVA, n = 3 biologically independent replicates per concentration and time point). In **(B)** the line shows the result of a log-logistic dose-response model adjusted to estimate the effective dose to trigger effects in 50% (ED50) of the individuals (n = 5 biologically independent replicates per concentration). See **Supplementary Datasheet 1** for detailed results of statistical analyses.

Earlier studies observed dosage-dependent effects of DNA on immune signals and seedling growth and revealed certain species-specific differences in the timing of the responses. Therefore, we first characterised the time course of H_2_O_2_ levels after treatment with different concentrations of self-DNA. We observed that H_2_O_2_ levels started to increase at 10 min, reached peak values at 15 min and returned to a base level at 60 min after treatment with 5 µg*·*ml*^-^* ^1^ DNA but not 50 µg*·*ml*^-^* ^1^ DNA (**Figure 1A**). Both DNA concentration and time after treatment had highly significant effects on H_2_O_2_ levels that were subject to a significant interaction (**Figure 1A**, see **Supplementary Datasheet 1** for details). Based on these results, 15 min after treatment was selected as a standard sampling time for H_2_O_2_ quantification in all subsequent experiments.

In a second experiment, the threshold concentration of DNA required to trigger a significant ROS response was determined. Self-DNA was applied at the beforementioned concentrations and additionally at 0.5, 0.05 and 0.005 µg*·*ml*^-^* ^1^ and H_2_O_2_ levels were quantified 15 min later (n = 5 independent biological replicates for each concentration, **Figure 1B**). Using a log-logistic dose-response model we determined 0.74 and 5.15 µg*·*ml*^-^* ^1^ as the effective doses that cause 50% and 99% responses (ED50 and ED99) respectively (**Supplementary Datasheet 1**). Based on these data, we selected 5 µg*·*ml*^-^* ^1^ of DNA as a standard concentration for all subsequent experiments.

### 3.2 Self/nonself DNA specific induction of H_2_O_2_ and defence hormones

**Figure 2:**
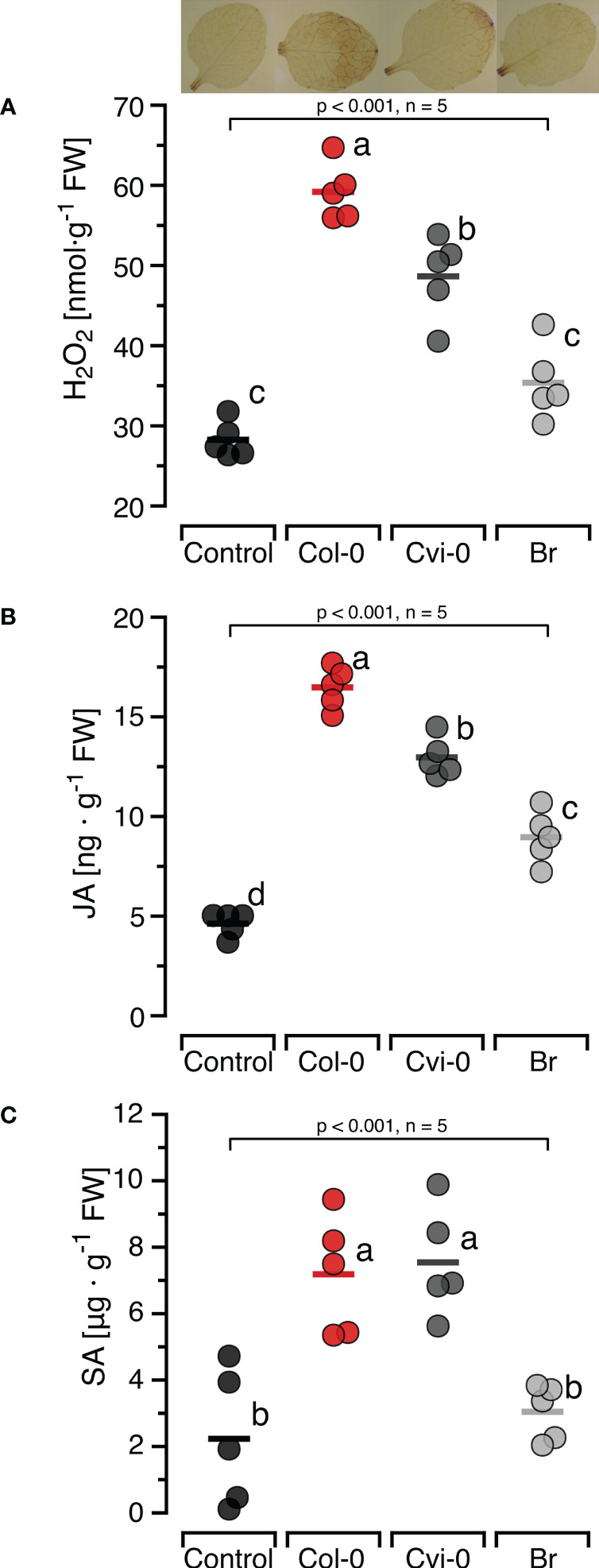
Self-nonself-specific induction of H_2_O_2_ and defence hormones by exogenous DNA. The levels of ROS [in nmol H_2_O_2_ g^-1^ leaf fresh weight], jasmonic acid [in ng JA g^-1^ leaf fresh weight] and salicylic acid [in µg SA g^-1^ leaf fresh weight] were determined at 15 min (H_2_O_2_, **A**), 30 min (JA, **B**) and 24 h (SA, **C**) after treating *Arabidopsis thaliana* Col-0 plants with 5 µg*·*DNA ml*^-^* ^1^ in 0.05% v v^−1^ Tween 20 of self-DNA (red symbols), nonself-DNA from *A*. *thaliana* ecotype Cape Verde islands (Cvi-0, dark grey symbols), or nonself-DNA from broccoli (Br, *Brassica oleracea*, light grey symbols). Controls (black symbols) were treated with 0.05% v v^−1^ Tween 20 in water. In all three panels, circles represent individual data points, horizontal lines indicate means, and different letters indicate statistically significant differences among plants treated with DNA from different origins (p < 0.05, *post hoc* Tukey tests). The p-values shown in the figure indicate the treatment effect on H_2_O_2_, JA and SA, according to separate one-way ANOVAs, n = 5 biologically independent replicates). See **Supplementary Datasheet 1** for detailed results of statistical analyses.

To confirm the self/nonself specific effects of DNA on H_2_O_2_ formation previously reported in other models and investigate whether this specificity also applied to the induction of the two major defence hormones, we first characterised the induction of JA and SA by self-DNA over time (**Supplementary Figure S3**) and selected 30 min as the sampling time for JA and 24 h for SA. Subsequently, we treated Col-0 plants with self-and nonself-DNA. DNA treatment had a significant and species-specific effect on the levels of H_2_O_2_, JA and SA at the respective sampling time (**Figure 2**). *Post hoc* Tukey analysis revealed that JA was induced by DNA from all three species, although with species-specific differences, while H_2_O_2_ and SA were induced by self-DNA and by nonself-DNA from *A. thaliana* Cvi-0, but not by broccoli DNA (**Figure 2**).

### 3.3 *In situ* damage generates ROS-inducing DNA fragments

**Figure 3:**
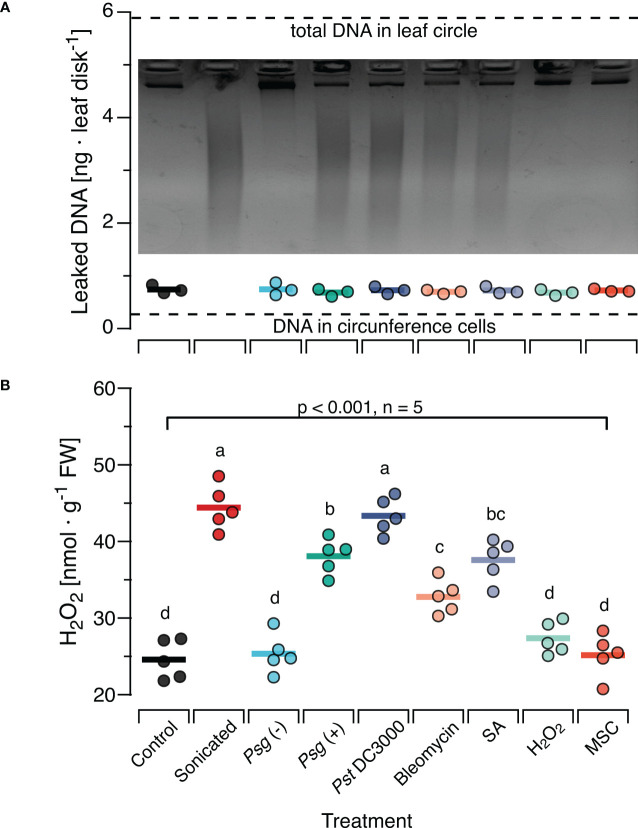
*In situ* damage by virulent bacteria or DNA-damaging molecules generates fragments with ROS-inducing activity. *Arabidopsis thaliana* Col-0 plants were pre-treated with pathogenic bacteria or DNA-damaging molecules and the DNA retrieved from leaf discs was analysed by gel electrophoresis for fragmentation **(A)** and for ROS-inducing activity **(B)**. Pre-treatments were inoculation with a suspension of 10^8^ bacterial cells of: *Psg* (–), avirulent *Pseudomonas syringae* pv. glycinea carrying vector pVSP61; *Psg* (+), virulent *Pseudomonas syringae* pv. glycinea carrying vector pV288 with effector avrRpt2; *Pst* DC3000, *Pseudomonas syringae* pv. tomato DC3000; or infiltration with 100µl of: Bleomycin, 0.6 µg ml^-1^ of bleomycin; SA, 100 µM salicylic acid; H_2_O_2_, 80 mM hydrogen peroxide. Control, leaf disks from plants with no prior treatment, MSC, mechanical stress control. **(A)** The mean amount of DNA [ng DNA per leaf disc] retrieved from 10 discs pooled from at least three individual plants is shown as circles that represent individual data points. We detected no significant effect of the pre-treatment on the amount of retrieved DNA (p = 0.908 according to one-way ANOVA, n = 3 biologically independent replicates). Dotted lines represent the estimated quantity of DNA in the cells of the circumference and the entire leaf disc, respectively. The photo shows a 2.2% agarose gel stained with ethidium bromide that was used to visually analyse the DNA for fragmentation. **(B)** The levels of ROS [in nmol H_2_O_2_ g^-1^ leaf fresh weight] were determined 15 min after treating Col-0 plants with a solution of 5 µg*·*DNA ml*^-^* ^1^ in 0.05% v v^−1^ Tween 20 of the retrieved DNA. No additional treatment for fragmentation was used except for the sonicated DNA that served as a positive control. In both panels, circles represent individual data points, horizontal lines indicate means, and different letters in **(B)** indicate statistically significant differences among treatments (p < 0.05, *post hoc* Tukey tests). The p-value shown in **(B)** indicates the treatment effect on H_2_O_2_ according to one-way ANOVA, n = 5 biologically independent replicates. See **Supplementary Datasheet 1** for detailed results of statistical analyses.

To investigate whether the effects of sonicated DNA on H_2_O_2_ levels are comparable to those elicited by naturally damaged DNA, we treated Col-0 plants with each of three bacterial pathogens or with DNA-damaging agents and collected the complete (fragmented and non-fragmented) DNA from subsequently excised leaf discs. This method yielded on average 0.8 ng DNA per leaf disc, with no statistically significant differences among treatments (**Figure 3A**). Based on our estimation, the total amount of DNA would have been ca 5.8 ng in the entire disc and 0.03 ng in the circumference (dotted lines in **Figure 3A**). Gel-electrophoresis of the recovered DNA revealed a strong fragmentation of DNA in leaves that had been inoculated with either of the two virulent bacterial strains, *Pst* DC3000 and *Psg* (+) or infiltrated with Bleomycin or SA. An analysis with Image Lab (Biorad) confirmed that most of the fragments fell within a size range of < 1000 bp, in the same range as in sonicated DNA. In contrast, we retrieved significantly lower levels of fragmented DNA from leaves inoculated with the nonvirulent *Psg* (–) strain or infiltrated with H_2_O_2_ and from non-treated leaves subjected to the mechanical stress control (Inset in **Figure 3A**; **Supplementary Figure S4**).

In a subsequent experiment, we used this DNA to treat Col-0 plants. We sampled leaves 15 min later and observed a highly significant effect of the previous treatment on the H_2_O_2_-levels in the DNA-treated leaves (p < 0.001, One-Way ANOVA, n = 5 biologically independent replicates, see **Supplementary Datasheet 1** for details). We detected a significant induction of H_2_O_2_ by DNA from *Pst* DC3000 infected or *Psg* (+) infected plants and by DNA from plants previously infiltrated with Bleomycin or SA (p < 0.05, *post hoc* Tukey tests, n = 5 biologically independent replicates, **Figure 3B**, see **Supplementary Datasheet 1** for details). In fact, we found no statistically significant difference (p =0.99) between the H_2_O_2_-inducing effect of DNA from *Pst* DC3000- infected leaves and of sonicated DNA. In contrast, DNA from plants inoculated with the avirulent *Psg* avrRpt2+, infiltrated with H_2_O_2_ or from mechanically damaged leaves had no statistically significant effect on H_2_O_2_ levels (p =0.99, p = 0.71, p = 0.99, respectively).

### 3.4 Effects of DNA on H_2_O_2_, defence hormones and resistance in the *atm* and *atr* mutant lines

**Figure 4:**
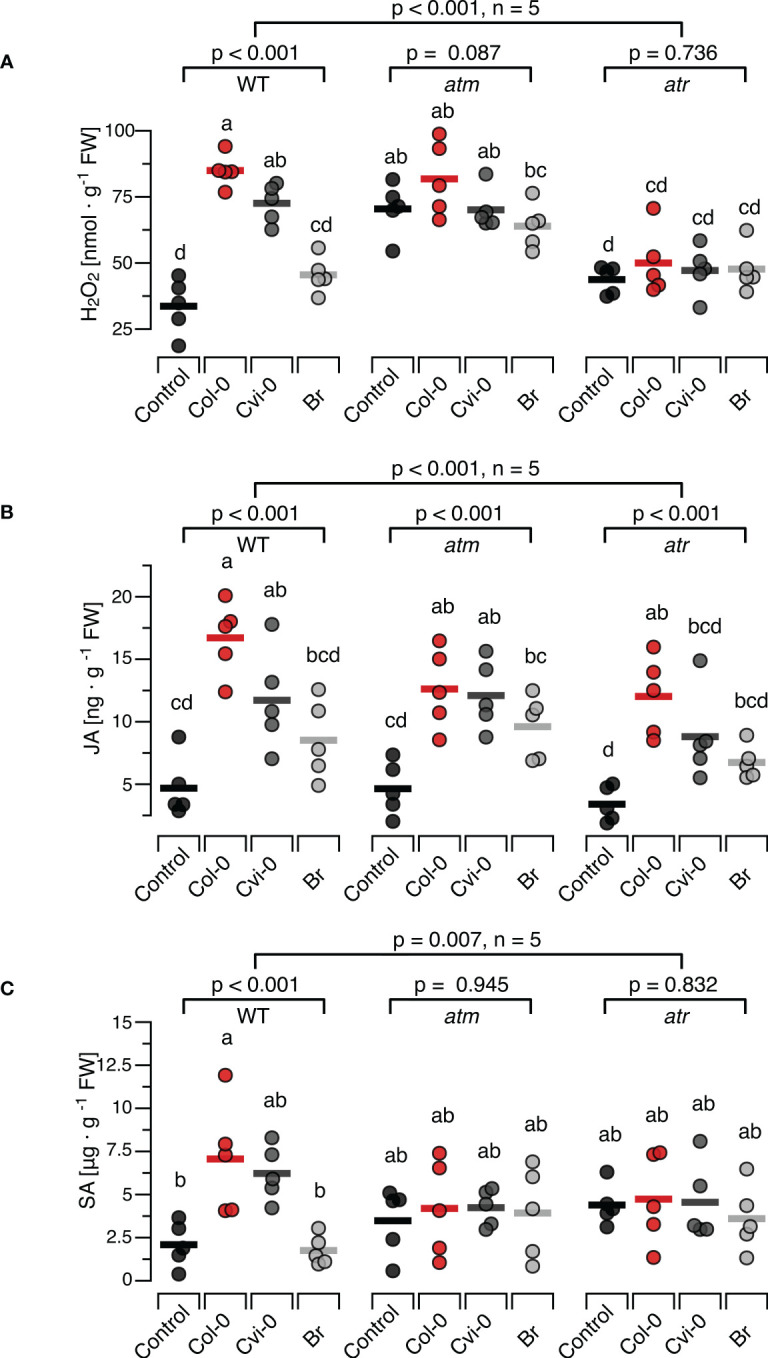
Arabidopsis DDR mutant lines *atm* and *atr* show self/nonself-specific JA induction by DNA but no induction of H_2_O_2_ and SA. The levels of ROS [in nmol H_2_O_2_ g^-1^ leaf fresh weight], jasmonic acid [in ng JA g^-1^ leaf fresh weight] and salicylic acid [in µg SA g^-1^ leaf fresh weight] were determined at 15 min (H_2_O_2_, **A**), 30 min (JA, **B**) and 24 h (SA, **C**) after treating *Arabidopsis thaliana* Col-0 wildtype (WT) plants or T-DNA insertion lines of Ataxia Telangiectasia Mutated (ATM) and ATM AND RAD3-RELATED (ATR) with a solution of 5 µg*·*DNA ml*^-^* ^1^ in 0.05% v v^−1^ Tween 20 of self-DNA (red symbols), nonself-DNA from *A*. *thaliana* ecotype Cape Verde islands (Cvi-0, dark grey symbols), or nonself-DNA from broccoli (Br, *Brassica oleracea*, light grey symbols). Controls (black symbols) were treated with 0.05% v v^−1^ Tween 20 in water. Circles represent individual data points, horizontal lines indicate means, and different letters indicate statistically significant differences among plants treated with DNA from different origins (p < 0.05, *post hoc* Tukey tests, n = 5, see **Supplementary data sheet 2** for detailed results of statistical analyses). The p-values shown in the figure indicate the treatment effect on H_2_O_2_, JA and SA, according to separate two-way ANOVAs, and according to separate one-way ANOVAS for each genotype (n = 5 biologically independent replicates in all cases, see **Supplementary Datasheet 1** for details).

To investigate whether central steps of the DNA-damage response are involved in induction of H_2_O_2_, JA or SA by exogenously applied DNA, T-DNA insertion lines *atm-1* (SALK_040423C) and *atr-2* (SALK_032841C) confirmed *via* genotyping to be homozygous (**Supplementary Figure S5**). We treated these plants with self and nonself-DNA from the before mentioned three sources, quantified H_2_O_2_ levels 15 min later and observed significant differences between the responses of the mutants and the wild type plants (p < 0.001 for DNA treatment, genotype and the treatment genotype interaction, Two-Way ANOVA, n = 5 biologically independent replicates, see **Supplementary Datasheet 1** for details). We could detect no statistically significant effects of DNA treatment on H_2_O_2_ levels in the *atm* or the *atr* plants (p = 0.087 and 0.736, respectively, One-Way ANOVA, n = 5 biologically independent replicates, see **Supplementary Datasheet 1** for details). Intriguingly however *atm* plants but not *atr* plants, were characterized by higher baseline H_2_O_2_ levels than WT plants (**Figure 4A**). In fact, Tukey *post hoc* tests revealed that the H_2_O_2_ levels in the *atm* plants, including untreated controls, were significantly higher than in WT control plants and not statistically different from self-DNA treated WT plants. In contrast, we could not detect any statistically significant differences when comparing the H_2_O_2_ levels in untreated WT control plants with those in *atr* plants in any of the treatment groups (**Figure 4A**; **Supplementary Datasheet 1**).

Whereas the ROS response to DNA in *atr* and *atm* mutants clearly differed from the response in WT, we observed a seemingly normal induction of JA by exogenously applied DNA in the mutants (**Figure 4B**). In fact, we observed a highly significant treatment effect on JA in the entire dataset and also when analysing each genotype with individual ANOVAs, while we detected only marginally significant differences among the mutants and the WT (**Figure 4B**, p = 0.012 for genotype and p = 0.453 for the interaction). By contrast, none of the mutants showed a detectable induction of SA (**Figure 4C**, p = 0.945 for *atm* and P = 0.832 for *atr*). In fact, even with *post hoc* tests comparing the SA levels in *atm* or *atr* plants of any of the treatment groups with those in untreated controls of the WT, we detected no significant differences (**Figure 4C**: p > 0.05 in all cases, see **Supplementary Datasheet 1** for details).

**Figure 5:**
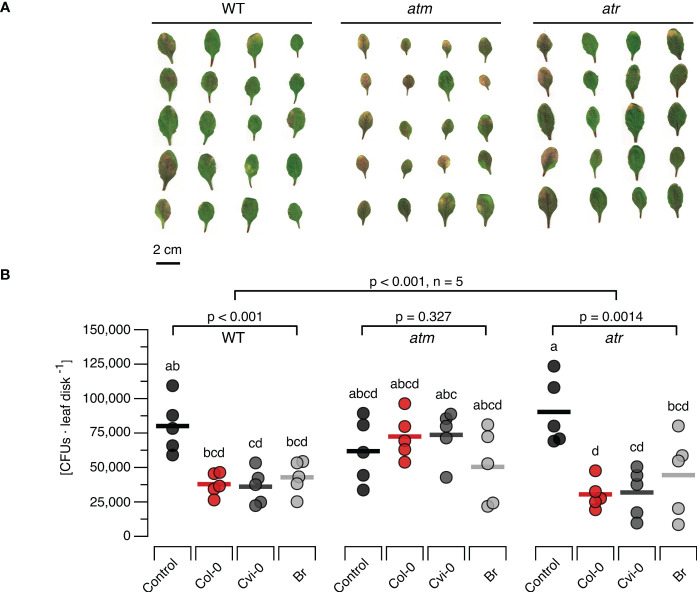
Arabidopsis DDR mutant line *atm* but not *atr* is affected in the immunity to a bacterial pathogen. Disease symptoms like chlorosis (yellowing due to the loss of chlorophyll) and necrosis (browning due to premature cell death) **(A)** and bacterial density [in colony forming units, CFUs, per leaf disc] **(B)** are shown seven days after inoculating *Arabidopsis thaliana* Col-0 wildtype (WT) plants or T-DNA insertion lines of Ataxia Telangiectasia Mutated (ATM) and ATM AND RAD3-RELATED (ATR) with a suspension of 10^8^ bacterial cells of *Pseudomonas syringae* pv. tomato DC3000. Inoculation was performed two days after treating the plants with a solution of 5 µg*·*DNA ml*^-^* ^1^ in 0.05% v v^−1^ Tween 20 of self-DNA (red symbols), nonself-DNA from *A*. *thaliana* ecotype Cape Verde islands (Cvi-0, dark grey symbols), or nonself-DNA from broccoli (Br, *Brassica oleracea*, light grey symbols). Controls (black symbols in **B**) were treated with in 0.05% v v^−1^ Tween 20 in water 2 days before inoculation. Circles in **(B)** indicate individual data points, horizontal lines indicate means, and different letters indicate statistically significant differences among plants treated with DNA from different origins (p < 0.05, *post hoc* Tukey tests). The p-values in **(B)** indicate the treatment effect on CFU numbers according to two-way ANOVA and according to separate one-way ANOVAS for each genotype (n = 5 biologically independent replicates in all cases, see **Supplementary Datasheet 1** for details).

To study the effects of DNA treatment on the resistance to bacterial pathogens we treated WT, *atm* and *atr* plants with each of the three types of DNA and challenged them 48 h later with *Pst* DC3000. Common disease symptoms like chlorosis (yellowing due to the loss of chlorophyll) and necrosis (browning due to premature cell death) were recorded and determined visually (**Figure 5A**) and bacterial densities were quantified as CFUs (**Figure 5B**) seven days post infection. In WT plants previously treated with different sources of DNA we could observe mild severity of both chlorosis and necrosis in comparison to the control treatment. The *atm* and *atr* mutants suffered from slightly stronger symptoms (a mix of chlorosis and necrosis symptoms) than the wildtype. However, while *atm* mutants previously treated with DNA still showed clear disease symptoms independently of the type of DNA, DNA treatment of *atr* mutants strongly reduced the severity of the symptoms, similar to levels as seen in treated WT. In summary, the evaluation of disease symptoms revealed that DNA-treatment triggered a significant resistance induction that did not depend on the source of the DNA in WT and *atr* plants, but not in *atm* plants. The overall analysis of CFU numbers confirmed a highly significant treatment effect (p < 0.001) but marginally significant effects of the genotype (p = 0.021) with a significant (p = 0.002) interaction. Moreover, individual ANOVAs confirmed a significant effect for the WT and for *atr*, while no significant effect of DNA treatment on CFU numbers could be detected for *atm* (p = 0.327, **Figure 5B**). Intriguingly, *post hoc* tests did not allow to detect significant differences among WT or *atr* plants treated with the different types of DNA and thereby confirmed that DNA induces resistance to *Pst* DC300 in a self/nonself-independent way (**Figure 5B**).

### 3.5 H_2_O_2_ -inducing properties are maintained in synthetic DNA and DNA with different CpG content

**Figure 6:**
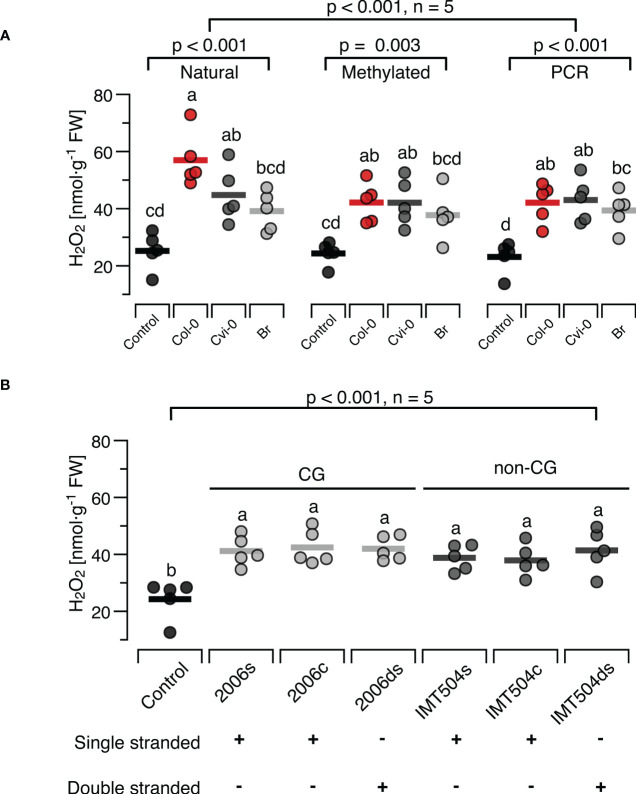
Presence and methylation of CpG motifs have minor effects on ROS-inducing properties of DNA. The level of ROS in [nmol H_2_O_2_ g^-1^ leaf fresh weight] was determined at 15 min after treating *Arabidopsis thaliana* Col-0 plants with 5 µg*·*ml*^-^* ^1^ DNA with different degrees of CpG methylation **(A)** or with synthetic oligodeoxynucleotides (ODNs), the CpG-containing 2006 and the CpG-free IMT504 **(B)**. In **(A)**, self-DNA from Col-0 plants (red symbols), nonself-DNA from *A*. *thaliana* ecotype Cape Verde islands (Cvi-0, dark grey symbols) or nonself-DNA from broccoli (Br, *Brassica oleracea*, light grey symbols) was used in its ‘natural’ state (only sonicated), after complete cytosine methylation in the CpG motif using the methyltransferase M.SssI, or served as template for genome amplification using 15-mer random primers. In **(B)** the CpG-containing ODN 2006 (5’- TCGTCGTTTTGTCGTTTTGT-3’) and the CpG-free ODN IMT504 (5’-CATCATTTTGTCATTTTGTCATT-3’) as well as their complementary sequence (2006c and IMT504c) were applied either as single-stranded (ss)ODNs or as annealed double stranded (ds)ODNs. Different letters indicate statistically significant differences (p < 0.05, *post hoc* Tukey tests, n = 5 biologically independent replicates in all cases). The p-values in **(A)** indicate the treatment effect on according to two-way ANOVA and according to separate one-way ANOVAS for DNA of each methylation status, and in **(B)** the treatment effect according to one-way ANOVA (n = 5 biologically independent replicates in all cases, see **Supplementary Datasheet 1** for details).

In the mammalian immune system, TLR9 binds preferentially to DNA that is rich in unmethylated CpG motifs while all other dsDNA sensors known so far bind DNA in a sequence-independent way (6, 7, 64, 109). Since the few studies that tested for a role of the unmethylated CpG motif in the immune response of plants show inconclusive results, we aimed at providing further evidence, by taking two independent approaches. First, we compared the H_2_O_2_-inducing properties of DNA with completely methylated CpG motifs (generated from natural self-or nonself-DNA that was treated with M.SssI, **Supplementary Figure S2**) versus completely unmethylated DNA (produced by a PCR with 15-mer random primers from natural DNA of all three species as templates). In both cases, the manipulated DNA had a significant effect on H_2_O_2_ levels, but with no detectable differences between DNA produced from the three different sources (**Figure 6A**). In fact, the treatment effect was significant in the complete experimental design and also when analysing the effects of natural DNA, methylated DNA and PCR-generated DNA separately, but *post hoc* tests identified significant differences only between controls and DNA-treated plants, while we could detect no significant effects for comparisons among methylated DNAs from different sources (p > 0.99, **Figure 6A**, see **Supplementary Datasheet 1** for details).

In the second approach we used the CpG- containing ODN 2006 and the CpG-free ODN IMT504 and observed a significant effect on H_2_O_2_ levels (**Figure 6B**). However, the H_2_O_2_-inducing effect was independent of whether we applied the ODNs or their complementary sequence (IMT504c and 2006c) either as single-stranded (ss)ODNs or as annealed double stranded (ds)ODNs. In fact, *post hoc* tests identified only the differences between the control condition and the treated plants to be significant, but they did not show any significant differences between the different treatments (p > 0.85 for all pairwise comparisons, **Figure 6B**).

## 4 Discussion

### 4.1 Plant DNA triggers self/nonself-specific activation of immune signalling in *Arabidopsis*

The accumulation of fragmented DNA in the cytoplasm or the extracellular space is a signal of danger. Mammals and plants respond to this danger with an activation of innate immunity. Our present study confirms earlier reports that plants exhibit a self/nonself-specific response to DNA from related species at a particularly fine taxonomic resolution: *A. thaliana* plants of ecotype Col-0 responded to treatments with self-DNA from other Col-0 plants with significantly stronger increases in the levels of H_2_O_2_ and JA than after treatments with nonself-DNA from another ecotype of the same species (**Figure 2**). In general terms, an immune response to ‘self’ contradicts the classical immunological paradigm as expressed in the title of Charles Janeway’s seminal publication “The immune system evolved to discriminate infectious nonself from noninfectious self” (110) but rather, provides strong empirical support for Polly Matzinger’s statement “The immune system is more concerned with entities that do damage than with those that are foreign” (111). Both perspectives provided the framework for important immunological breakthroughs, from the discovery of PRRs guided by the Janeway paradigm to providing the explanation of pro-inflammatory self-molecules as DAMPs, which were predicted by Matzinger and discovered empirically by Walter Land in 1994 (112, 113) and since then are increasingly being identified as drivers of important beneficial as well as detrimental functions of the immune system (44, 114, 115).

Our knowledge on the mechanisms that control DAMPs-triggered plant immunity is still very limited and few receptors have been identified so far (41, 116–118). Considering the opportunities provided by the mutants and multiple other genetic and molecular tools existing for *Arabidopsis thaliana* for future research into response to DNA we characterised the dose-response relations and the time course the ROS-response to self-DNA, and identified 5µg DNA ml^-1^ as a concentration that in 99% of cases should induce a significant H_2_O_2_ accumulation that reaches peak – and likely maximum – levels at 15 min (**Figure 1**). The levels of JA peaked at 30 min and those of SA at 24 h (**Figure 2**; **Supplementary Figure S3**). Subsequent treatments revealed self/nonself-specific effects of DNA on H_2_O_2_ and both hormones and in all cases, strongest effects were triggered by self-DNA (**Figure 2**), similar to earlier observations, although with minor differences between species (20–22, 26, 35, 36). The response times and levels reached by the three signals fall clearly within the standard kinetics of the three signals (119–121).

### 4.2 Infection and DNA-damaging agents generate ROS-inducing DNA fragments

In spite of numerous reports that associate infection or herbivore feeding with damage to the host’s DNA, it remains unknown whether the release of self-DNA fragments is a common outcome of an attack by herbivores or pathogens. Moreover, doubts remained as to which degree the exogenous application of sonicated plant DNA causes effects that are comparable to those when DNA is damaged *in situ* in more realistic scenarios. Therefore, we aimed to compare the ROS-inducing properties *in situ*-damaged DNA to the effects of sonicated DNA.

To this end, we challenged Col-0 plants with various pathogenic bacteria or treated them with H_2_O_2_, SA or bleomycin (a radiomimetic drug that triggers DSBs (94, 122, 123)). Aiming to collect only DNA that had leaked into the apoplast we followed a method developed to study extracellular NAD+ a SAR-triggering DAMP (96). The results revealed the presence of massive amounts of DNA fragments in a size range between ca 100 bp and 1000 bp obtained from leaves previously infected with *Pst* DC3000 or *Psg* (+) or treated with Bleomycin or SA. In contrast, infection with *Psg* (–) and treatment with H_2_O_2_ did not cause major fragmentation of plant DNA, at least in terms of generating visible amounts of fragments in our assay. For the bacteria, this pattern confirms earlier reports that characterised the first two strains as virulent bacteria that inflict intensive damage to their host´s genomic DNA, whereas *Psg* (–) exhibits a very low level of virulence although it can reproduce in Arabidopsis (92, 93, 124, 125). Similarly, our results confirm for bleomycin and SA, but not H_2_O_2_, that the *in situ* effects of widely used DNA-damaging agents comprise the generation of fragments within the same size range as sonication. At the first glance, the lack of massive DNA fragmentation upon H_2_O_2_, treatment seems counterintuitive. While the role of SA as a DNA-damaging agent that had been proposed by the group of Xinnian Dong (59) has later been questioned by a study that reported damage-induced SA to be involved in subsequent repair (93), the role of oxidative stress as a DNA-damaging factor can be considered as generally accepted (126–128). However, studies using a wide variety of experimental models from the plant and animal kingdom identify SA and H_2_O_2_ as ‘double-edged swords’ that act as DNA damaging agents but also serve as signals to induce adequate countermeasures (129–132). Considering that most of the involved processes are dosage-dependent, follow different kinetics and are likely to interact *via* diverse direct and indirect mechanisms, it should not come as a surprise that the net outcome quantified at any single time point yields seemingly contrasting results.

Originally, the motivation for this experiment was to demonstrate the leakage of DNA fragments from intact cells into the apoplast. We reasoned that our method would allow the recovery of leaked DNA from most cells in the leaf disc, whereas in a scenario in which DNA release requires mechanical disruption of cells, only DNA from cells in the circumference would be retrieved. To this end, we estimated the total amount of DNA in the leaf disc versus the circumference. Comparison of the average amount of DNA retrieved from each leaf disc to these hypothetical values indicates that the retrieved DNA had most likely had been released only from the cells in the circumference that were mechanically damaged when cutting out the leaf discs. Thus, further work will be required to provide unambiguous evidence for the release of DNA fragments from stressed but still intact cells. More importantly, a mechanical stress control based on application of gentle pressure on leaf discs from untreated plants during the DNA elution process clearly demonstrates that mechanical stress during the collection procedure did not generate detectable amounts of DNA fragments (last lane, **Figure 3A**; **Supplementary Figure S4**). Treatments of Col-0 plants with the collected DNA confirmed that infection with virulent bacteria or DNA-damaging agents generates fragments of DNA with similar H_2_O_2_-inducing properties as those of sonicated DNA (**Figure 3B**).

### 4.3 TLR9-like mechanisms of DNA recognition do not explain self/nonself-specific effects in plants

Based on this validation of sonicated DNA as an experimental model, it seems safe to conclude that plants exhibit stronger immune responses to self-DNA than to nonself-DNA from closely related sources. This specificity resembles the effects of leaf homogenates, which arguably contain a cocktail of DAMPs (86) and thereby, supports a role of self-DNA as DAMP (133). Unlike mammals, which express several dsDNA receptors in different subcellular compartments, no dsDNA receptors are known from plants. Evidently, a lack of published evidence does not exclude the possibility that plant dsDNA sensors simply remain to be discovered. Nevertheless, it appears difficult to envision receptors able to differentiate between the fragmented genomes of two closely related plant species. For mammals, the differential activation of murine versus human plasmacytoid Dendritic Cells (pdCs) by single-stranded RNA of Human Immunodeficiency Virus Type 1 (HIV-1) could be attributed to TLR 7 and 8 (134) and most recently, species-specific differences have been discovered among the mechanism used for DNA binding by cGAS from different mammalian species (46). Still, the only mammalian dsDNA sensor known to distinguish DNA from organisms belonging to different phylogenetic groups seems to be TLR9, which binds preferably DNA that is rich in unmethylated CpG motifs and thus allows for differential immune responses to bacterial and viral DNA versus genomic DNA of eukaryotes (135–137), i.e., a differentiation at the level of domains.

This particular feature of TLR9 motivated earlier studies to test for a role of CpG motifs and/or cytosin methylation in the plant immune response to DNA. Yakushiji and colleagues (9) observed that the accumulation of ROS in Arabidopsis in response to genomic or plasmid bacterial DNA decreased after methylation of the cytosine residues in the 5’-CG-3’ sequence using the DNA methyltransferase M.SssI (99). Moreover, the authors used the same methyltransferase in combination with restriction enzymes to corroborate the methylation of natural herring DNA and thereby could attribute the lower ROS-inducing activity of herring DNA to a TLR9-like mechanism that requires unmethylated CpG motifs (9). Similarly, a recent study using the same three enzymes to generate DNA free of unmethylated CpG motifs from naturally methylated DNA of common bean and observed that any enzymatic manipulation decreased the H_2_O_2_-inducing properties as compared to the natural self-DNA, with no detectable differences among fragments with completely methylated CpG motifs and fragments in which all unmethylated CpG motifs had been cleaved enzymatically (36). These results would be consistent with a mechanism in which DNA triggers a partial induction of H_2_O_2_ that is independent of CpG-methylation whereas the full response requires additional activation by unmethylated CpG motifs (36). However, another recent study (23) discovered that treatments with the CpG containing ssODN 2006 and the CpG-free ssODN 504 triggered various immune responses in Arabidopsis, including H_2_O_2_ formation and phenotypic resistance to a fungus and the bacterial pathogen *Pst* DC3000 (**Table 1**), again with no detectable differences between the two ODNs (23).

In the present study we observed a reduction in the H_2_O_2_-inducing effect after M.SssI-mediated methylation that eliminated the self/nonself-specific differences among DNA from both Arabidopsis ecotypes and from broccoli (**Figure 6A**).We also observed an induction of H_2_O_2_ when using synthetic – and thus, completely unmethylated - DNA that was statistically not different from the levels observed after treatments with completely methylated DNA, but quantitatively lower than the level triggered by natural self-DNA (**Figure 6A**). Furthermore, we repeated the crucial experiment reported by Toum and colleague (23) and observed that synthetic ODNs induced H_2_O_2_ to levels that were similar to those triggered by completely methylated or complete unmethylated DNA, and with no detectable differences between CpG-containing and CpG-free or single-stranded versus double-stranded ODNs (**Figure 6B**).

We consider it difficult to envision a straightforward explanation of these results within a framework that is exclusively based on a TLR9-like recognition of unmethylated CpG motifs and conclude that other properties of natural plant DNA – perhaps the plant-specific methylation in CHG and CHH motifs – could contribute to the full immunogenic effects of self-DNA. The proposed role of methylation in CHG and CHH would be consistent with the induction of SA to similar levels by Col-0 and Cvi-0 DNA (**Figure 2C**; p = 0.985 for the difference between the sources, see **Supplementary Datasheet 1** for details). The Arabidopsis ecotype Cvi-0 is well known for having the lowest degree of CpG methylation in genes bodies among more than 1000 *A. thaliana* ecotypes, but Cvi-0 and Col-0 have very similar global methylation patterns in CHG and CHH motifs (138). Evidently, future work will be required to confirm the suggested role of methylation in non-CpG motifs and even if confirmed, it would remain to be verified if the minor differences between Col-0 and Cvi-0 shown suffice to explain the significantly different effects on H_2_O_2_ and JA.

### 4.4 ATM and ATR are required for SA induction by exogenous DNA fragments

Taken together, the beforementioned observations open promising perspectives, but they do not present a basis to exclude that a process connected to the DDR is contributing to the activation of immune signalling by extracellular DNA fragments. The DDR evolved for the repair of an organism´s own genome and thus, it seems plausible to assume that some of the involved elements work best for the own DNA but only partially when dealing with nonself-DNA; similar to the recently discovered differences among the mechanisms that control the self-DNA reactivity of cGAS molecules from different primates (46). We used mutant lines of *atr* and *atm* because both kinases are conserved among plants and mammals and well-known for their central role in the coordination of the DDR (139). While ATR is mainly activated by single-strand breaks, ATM is activated by double-strand breaks (DSB) and necessary for their repair, thus playing a crucial role for DNA repair *via* homologous recombination (47, 51, 62, 79, 80, 140). Moreover, a transcriptomic analysis of *atm* and *atr* mutants of Arabidopsis after exposure to γ-radiation revealed hundreds of upregulated genes, and the induction of ‘virtually all’ of these genes depended on ATM, but not ATR (79). Thus, it seems safe to assume that ATM should play a crucial role in DDR-related mechanisms that are activated during infection and thus, potentially linked to SAR induction.

Both mutants responded to DNA with a self/nonself-specific induction of JA that could not be distinguished from the response of the wild type (**Figure 4B**). The induction of JA *via* a DDR-independent mechanism should not come as a surprise: while links between SA and DNA damage have been reported in numerous studies for plants and mammals (59, 130, 132, 141–146), we are not aware of a study that liked the DDR to JA-signalling. In contrast, both mutants failed to respond to self- or nonself-DNA with a detectable induction of H_2_O_2_ or SA; at least, we could not detect any statistically significant effect of DNA the levels of H_2_O_2_ or of SA in any of the mutants (**Figures 4A, C**). *Post hoc* tests indicate that in the case of H_2_O_2,_ this lack of inducibility was likely due to increased baseline levels in *atm*, but not in *atr*. Correspondingly, *atm* plants exhibited a slightly increased basal level of resistance to *Pst* DC3000 (CFU numbers not statistically different from DNA-treated WT plants) but failed to induce resistance upon DNA treatment (no detectable treatment effect, **Figure 5B**). This observation is consistent with the assumption that an increased accumulation of unrepaired DNA damage in *atm* mutants triggers ongoing downstream signalling which ultimately exacerbates an efficient response to infection. Similarly, this scenario would be consistent with the observation that the *atm* and *atr* plants exhibited basal SA levels between basal levels and self-DNA-induced levels in WT. In fact, *post hoc* tests confirmed for the WT a significant induction of SA by self-DNA, but did not allow to detect a significant difference between the SA-levels in any of the *atm* or *atr* treatment groups and the SA levels in WT controls, but also when compared with the self-DNA induced SA levels (with three exceptions, p > 0.5 in all comparisons): thus, *post hoc* tests definitively place the basal SA level in the mutants quantitatively between the basal and the self-DNA inducible level in WT (See **Supplementary Datasheet 1**).

Surprisingly, though, *atr* plants exhibited a DNA-inducible resistance to *Pst* DC3000 that did not depend on the source of DNA and thus, resembled the pattern observed in wildtype. We lack a convincing explanation of this phenomenon. Resistance in Arabidopsis to *Pst* DC3000 is generally assumed to be under the control of SA. An SA-independent but JA-dependent signalling controls induced systemic resistance (ISR) triggered by Rhizobacteria and is mainly based on priming, rather than direct induction of resistance trait expression (147). Therefore, a similar, priming-based phenomenon might explain our observation.

### 4.5 DNA damage, DDR and SAR-induction – state of the art and open questions

Pathogen attack, mechanical damage and even sucrose treatment (a DAMP mimic triggering JA accumulation and JA-dependent defence activation in lima bean (148)) have been reported to induce nuclease activities and DNA cleavage in the host plant (149): a scenario that would link damage to DNA inflicted by DNases to the WR, rather than SA-mediated signalling. Likewise, the induction of ROS by ssODNs (23) and the lack of any differences among the responses to ssODNs and dsODNs (**Figure 6** of the present study) hardly fit into a classical DDR scenario.

Nevertheless, numerous observations link DNA damage and the DDR to the induction of SA-dependent SAR. For general reviews on these topics, we refer to recent reviews for different aspects of the DDR, of SA-mediated SAR and of the DAMPS-mediated SWR (4, 40, 41, 47, 51, 55, 69, 129, 133, 150, 151). Since the late 1960s, pharmacological studies reported on the resistance-inducing effects of DNA-damaging compounds (53, 54, 95, 152). More recently, a direct genetic connection among both processes has been demonstrated in studies that identified the transcription repressor SUPPRESSOR OF NPR1-1, INDUCIBLE 1 (SNI1) as part of the STRUCTURAL MAINTENANCE OF CHROMOSOME (SMC) complex (70) or reported that RAD51 induces SAR by replacing SNI1 from the promotor regions of PR1 (58, 59, 144). The SMC complex controls homologous recombination (59, 144), an error-free ATM-dependent (79) mechanism for the repair of DSB in which the RAD51 filament facilitates homology search and subsequent DNA strand invasion followed by DNA synthesis using the homologous strand as template (144). Homologous repair is essential for crossing over during meiosis (153), but it has also been reported repeatedly to occur at increased frequencies in pathogen-infected plants (57, 58, 154). The recruitment of ATM to DSB depends on the Meiotic Recombination 11(MRE11) RADIATION SENSITIVE 50 (RAD50) Nijmegen breakage syndrome 1(NBS1) (MRN) complex (47, 155). The processing of DSB by the MRN-complex depends on the nuclease activity of its elements, in particular MRE11, which leads to the formation of 3’ ssDNA overhangs (156, 157). These ssDNA overhangs explain why the activation of ATR upon DSBs requires ATM and the MRN complex (155, 158). Intriguingly, in the absence of NBS1, MRE11 and RAD50 localize to the cytoplasm. Therefore, MRE11 has been brought forward as a cytosolic sensor for dsDNA, at least in vertebrates. The role of NBS1 for the nuclear localization of the MRN complex and the subsequent recruitment and activation of ATM could be confirmed for Arabidopsis (140).

Nevertheless, doubts remain concerning the role of SA and concerning the role of SA-induced NPR1 in PR gene expression. SA has been brought forward as a DNA-damaging agent in a study published by the group of Xinnian Dong (59) while others found damage-induced SA to reduce damage (93). Therefore, Nisa and colleagues (51) consider the causal reasons of DNA damage during infection as ‘unknown’. Moreover, the beforementioned se observations would place RAD51 within the ATM-controlled mechanisms that are activated upon DSBs. However, Xinnian Dong´s group considers ATR/RAD17 as inducers of RAD51 (59, 144). RAD51 can either move to the site of DNA damage and facilitate homologous recombination or move to the PR-1 gene promotor to facilitate expression, either by replacing SNI1 or by binding to the promotor and thus trigger expression in an NPR1-independent pathway (144, 145). In honour of Xinnian Dong, we hereinafter use the term ‘Dong-pathway’ for the SA-independent induction of PR-1 gene expression by RAD51 which upon activation by ATR/RAD17 downstream to SA-inflicted SSB directly moves to the PR-1 promotor (58, 59), while in the presence of SA-activated NPR1, RAD51 can favour PR-1-expression by removing SNI1 from the PR-1 promotor (144).

In some cases, seemingly incongruent findings could reflect species-specific differences (159), mutants that generate changes in the phenotypic resistance levels *via* changes in the sensitivity to a certain signal rather than signal intensity (160), the context-dependent action of alternative mechanisms, e.g., during different parts of the cell cycle (79), different times of the day or different contributions of partly redundant elements. For example, plants possess two pathways for the synthesis of SA, the isochorismate-pathway in which ISOCHORISMATE SYNTHASE (ICS) catalyses the synthesis of the central precursor molecule and the PHENYLALANINE AMMONIA LYASE (PAL) pathway in which PAL, the first enzyme in phenylpropanoid synthesis, converts phenylalanine to trans-cinnamic acid (77). Although pathogen-induced SA synthesis has been considered to be mostly dependent on the ICS pathway (74, 78), this pattern has been questioned by others (72). Indeed, a study transforming Arabidopsis lines that overexpressed RAD51A from maize (*ZmRAD51A)* reported increased SA levels and observed RAD51 to be induced by SA and vice-versa. Moreover, the same plants exhibited a strong expression of PAL upon pathogen challenge – but not of ICS1 (161).

Similarly, an existence of both NPR1- dependent and NPR1-independent mechanisms of PR1 induction as proposed by the Dong group (58, 59) could result in scenarios attributing from ‘fine-tuning’ to ‘essential’ roles to SNI1 (69).

### 4.6 Fitting SAR induction by DNA fragments into the DDR: a working model

Even the NPR1-independent SAR induction *via* the ‘Dong-pathway’ requires SA as the DNA-damaging agent. Moreover, most studies assign partly overlapping, additive or even redundant functions to ATM and ATR (162, 163), mainly because both kinases signal through the shared SUPPRESSOR OF GAMMA RESPONSE 1 (SOG1) (80, 139, 164, 165). With exception of the sterility of double mutants of atm and atr (163), it seems difficult to envision a strictly ATM – and ATR-dependent process as it is indicated by our data for the DNA-induced increased in SA (**Figure 4C**).

**Figure 7:**
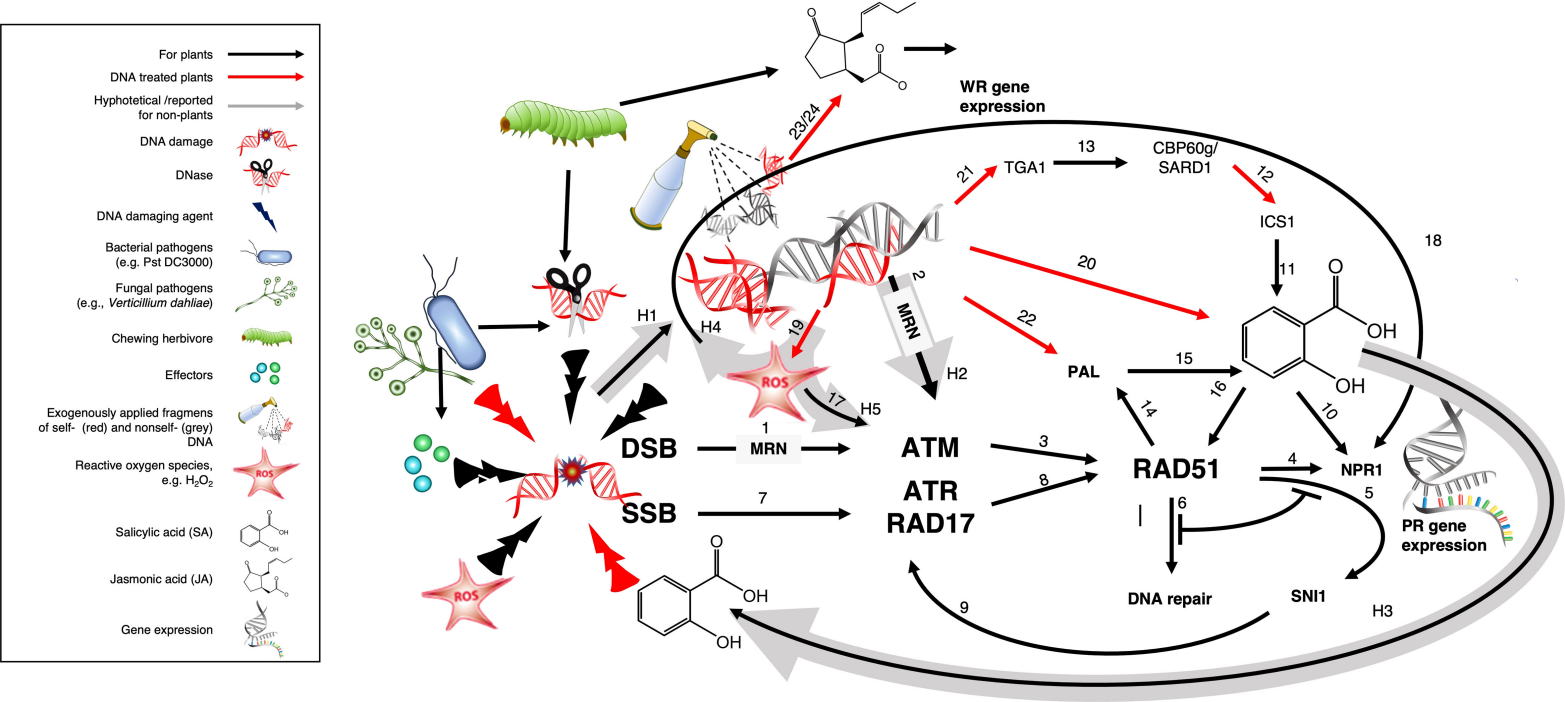
Graphical presentation of a working model fitting SAR induction by DNA fragments into the DDR. Exogenously applied DNA fragments could activate ATM *via* the MRN-complex to induce RAD51 and subsequently the ISOCHORISMATE SYNTHASE (ICS) and/or the PHENYLALANINE AMMONIA LYASE (PAL) biosynthetic pathway of salicylic acid (SA), which triggers DNA damage and subsequently, ATR, thereby connecting the ATM-based and NPR1-dependent pathway to the ATR-dependent and NPR1-independent Dong-pathway to PR1 expression. Functions corresponding to the arrows and supporting references are listed in **Table 3** according to the number used to identify the respective arrow. Black arrows indicate published evidence, red arrows highlight evidence from DNA-treated plants and grey arrows numbered H1-H5 indicate the different hypotheses underlying this model. See main text section ‘4.5. Fitting SAR induction by DNA fragments into the DDR: a working model’ for further references and explanations.

Based on the results of the present study and published information on resistance induction and the DDR (mainly – but not exclusively - in plants) we propose the following working model aimed at guiding the next steps in research (See **Figure 7** for a graphical representation, numbers of specific steps as cited in the following text in round brackets refer to the number used to identify the respective arrow in this Figure).

**Table 3:** Functions and supporting references corresponding to the arrows in the working model (**Figure 7**).

Arrow	Function	Reference
1	Double strand breaks (DSB) activate ataxia-telangiectasia mutated (ATM)	(47)
2	The MRE11/RAD50/NBS1 (MRN) complex identifies DSBs and binds to sites of DNA damage to subsequently recruit and activate ATM	(51, 140)
3	The induction of RAD51 is considered as ATM dependent	(79)
4	In the presence of NPR1, RAD51 can favour PR1-expression by removing SNI1 from the PR-1 promotor	(144)
5	Alternatively, RAD51 can move to the PR1 gene promotor to facilitate expression *via* an NPR1-independent pathway	(58, 59)
6	RAD51 can move to the site of DNA damage to coordinate repair	(59)
7	Single strand breaks (SSB) activate ATM- and Rad3-Related (ATR)	(47)
8	RAD17 and ATR activate RAD51 in response to salicylic acid (SA)-induced DNA damage to facilitate PR1 expression *via* the NPR1-dependent or independent pathway	(59)
9	ATR and RAD17 are DNA damage sensors under the negative control of suppressor of npr1-1, inducible 1 (SNI1) which forms part of the SMC5/6 complex	(59)
10	SA is crucial for SAR because binding of SA to cytoplasmatic NO-PATHOGENESIS-RELATED PROTEIN 1 (NPR1) oligomers induces the monomerization and translocation of NPR1 to the nucleus where monomeric NPR1 binds to the promoter region of PR1 to trigger SAR	(69, 74, 77, 166)
11	The synthesis of SA during infection depends mainly on the isochorismate pathway in which Isochorismate synthase (ICS1) catalyses the rate-limiting step.	(58, 74, 77)
12	ISC1 expression is under the control of the transcription factors SAR-DEFICIENT 1 (SARD1) and CALMODULIN BINDING PROTEIN 60g (CBP60g)	(58, 151, 167)
13	SARD1 and CPG60g are under positive control of TGACG-sequence-specific protein-binding (TGA) 1 transcription factors	(151)
14	A second SA biosynthetic pathway is the phenylalanine ammonia-lyase (PAL) pathway, PAL is induced by RAD51	(74)
15	PAL synthesizes the precursor of SA	(161)
16	SA induces RAD51. The feedforward loop 14-15-16 explains why RAD51 overexpressing plants exhibit increased baseline levels of SA	(145, 161)
17	Oxidative stress/H_2_O_2_ activate ATM	(168–171)
18	Increased levels of H_2_O_2_ are counterbalanced by an accumulation of antioxidants including GSH, thereby generating a more reducing environment. These redox changes downstream to ROS favour NPR1 monomerization formation and thereby activate NPR1 independently of SA	(69, 166)
19	Exogenous fragments of self- and nonself-DNA induce increased levels of H_2_O_2_, with self-DNA triggering stronger increases	(21) and present study **Figure 2**
H1	During infection or herbivore attack, pathogens, herbivores and the plant host itself secrete DNases, causing the generation of fragments of DNA that accumulate in the extracellular space	(168–170, 172)
H2	Exogenously applied and endogenous DNA fragments can activate ATM *via* the MRN complex	(173)
H3	SA can act as DNA-damaging agent independently of its synthetic origin (the PAL versus ICS pathway)	(59)
H4/H5	As alternative, exogenously applied fragments of self- and nonself-DNA could contribute to the activation of ATM and NPR1 *via* their ROS-inducing effect	
20	Exogenous fragments of self- and nonself-DNA induce SA	(36) and present study **Figure 2**
21	Exogenous fragments of self- and nonself-DNA induced the expression of TGA1, CBP60g and – likely in consequence - ICS1 in *A. thaliana*	(24) **Supplementary Table S2**
22	Exogenous fragments of self- and nonself-DNA induced PAL expression in *Lactuca sativa* and in *A. thaliana*	(22, 24) **Supplementary Table S2**
	Based on 20-22, we assume H2 to be more likely to explain the dependency of SA induction by exogenous DNA on ATM and ATR than H4/H5	
23	Exogenously applied fragments of self- and nonself-DNA induce jasmonic acid (JA), with self-DNA triggering stronger increases	(36) and present study **Figure 2**
24	Exogenously applied fragments of self- and nonself-DNA induced the expression of JA-biosynthetic genes	(24), see **Supplementary Table S2**

#### 4.6.1 Hypothesis

Processing of DSBs by the MRN complex generates – besides 3’ ssDNA overhangs – ssODNs, which can activate ATM independently of DSB, at least in vertebrates, as demonstrated by treatments with annealed oligonucleotides consisting of 70 bases of random complementary sequences or poly-dA70/poly-dT70 (pA70/pT70) (173). Assuming that DNases and other factors during infection or herbivore attack generate fragments of DNA that accumulate in the extracellular space (arrow H1) and assuming further, that these fragments can enter the cell and activate ATM via the MRN complex (H2), exogenously applied fragments of self- and nonself-DNA could activate ATM and thereby induce the synthesis of SA, which triggers DNA damage (H3) and thereby activates ATR (arrow 7) to close the circle by connecting ATM-based and NPR1-dependent PR1 expression to the ATR-dependent and NPR1-independent Dong-pathway (4–6).

In case that this mechanism existed in plants, it could be the pathway *via* which exogenously applied DNA fragments or DNA-damaging agents induce RAD51 expression and subsequent SA accumulation *via* a DDR-mediated pathway that depends on ATM and ATR. This assumption remains to be empirically supported, although the observation that *atmre11* mutant lines failed to respond to bleomycin treatment with increased expression of RAD51 provides some indirect support (123). More importantly, the proposed mechanism would be consistent with the complete failure of both mutant lines to exhibit a DNA-induced SA accumulation. Taking into account that the DDR is considered as independent of cytosine methylation (172), this scenario would also explain the responses to ssODNs and dsODNs as well to completely methylated versus unmethylated DNA.

As alternative scenario, ROS have been reported to activate ATM *via* an SA-independent pathway (17) (168–171). Moreover, following an initial oxidative burst, plant cells attain a more reducing environment due to the accumulation of antioxidants, such as reduced glutathione (GSH), which is considered to be one of the most important ROS scavengers (166, 174). Although glutathione-S-transferases perform a variety of pivotal functions, their most important effect is to catalyze peroxidase reactions to control oxidative stress (175). Increases in the ratio of reduced with oxidised glutathione were found to be associated with the increased appearance the reduced monomeric form of NPR1 monomers which then moves into the nucleus to control SAR-related gene expression (69, 166). Therefore, exogenously applied fragments of self- and nonself-DNA could contribute to the activation of ATM and NPR1 *via* their ROS-inducing effect Figures (20) (H4/H5). In fact, significant changes in the expression of numerous glutathione-S-transferases have been observed upon DNA treatment of Arabidopsis (**Supplementary Table S1**). Therefore, it seems possible that DNA fragments activate NPR1 *via* the induction of ROS and downstream redox changes.

However, self- and nonself-DNA trigger increases in SA in wild type Arabidopsis plants and common bean (this study and (35, 36)) and they induced ICS1 and ISC2, PAL, transcription factors controlling ICS1 and other central steps of SA synthesis (22, 25) (**Supplementary Table S1**). In addition, even fungal DNA induced PAL expression in Chili pepper plants (27). Considering this evidence for an induction of both pathways of SA biosynthesis (21, 22, 27) by exogenously applied DNA fragments and several earlier reports that identified the induction of PAL activity as crucial for resistance induction by DNA-damaging agents like actinomycin D or even by histone fragments (54, 95, 152, 176), we consider an activation of ATM *via* the MRN complex (H2) and subsequent SA synthesis as the more likely scenario.

If the MRN-dependent activation of ATM by extracellular DNA fragments was to be used as a model for studying the plant immune response to DNA, fragments of DNA that are generated during enemy attack should enter *via* the same pathway (H1). Pathogen-derived DNases have been brought forward repeatedly to link DNA damage to PR gene expression. Indeed, several pathogens and even herbivores of plants secrete DNases as effectors or as part of their nutrient acquisition strategy (177–182). The putative relevance of extracellular self-DNA fragments in the immunity of plants to biological enemies has gained empirical support by the recent discovery of extracellular DNases in the salivary secretion of the brown plant hopper (*Laodelphax striatellus*) and the fungal pathogen *Cochliobolus heterostrophus* (181, 182). However, the benefits of these DNases were attributed to a function of extracellular DNA as a DAMP in one study and as a direct defence mechanism in the other study, and we are not aware of direct evidence for a release of DNA fragments into the extracellular space in this context. Our first attempt to provide this evidence has, seemingly, failed (**Figure 3**). Therefore, the suggested role of H2 in the DDR-dependent plant immune response to DNA during natural enemy attack remains a hypothesis (H5) that requires further investigation.

### 4.7 Conclusions and outlook

Our present study validates the exogenous application of sonicated as a suitable model to study immune responses in plants to fragmented DNA and shows that Arabidopsis exhibits a highly self/nonself-DNA-specific induction of ROS and the defence hormones, JA and SA. We also consider our observations as a clear support of an involvement of ATM and ATR in the induction of SA by DNA. The recruitment of ATM and ATR to damaged DNA sites depends on the MRN complex and – although in DNA repair must take place in the nucleus – two elements of this complex are also expressed in the cytoplasm. In this scenario, a component of the MRN complex – most likely the nuclease MRE11 – would play the role of a cytoplasmatic DNA sensor in plants, as it has already been suggested for mammals (157).

To guide future investigation, we propose a model that – although as yet merely hypothetical – would be consistent with diverse results published by others and that offers a possibility how two seemingly contrasting roles of SA could be merged into a single mechanism. In this context, it seems worth to highlight that the induction of elements of both SA biosynthetic pathways by exogenously applied DNA validates our decision to quantify the defence hormones themselves as a more integrative outread, rather than focusing on the expression of pre-selected marker genes which in our case likely would have missed the induction of the PAL pathway by DNA. More importantly, our model is based on two assumptions that remain to be empirically supported and it does not explain the most interesting feature of the response. Considering the induction of numerous nucleases by DNA and the continuing discovery of new classes of nucleases (183), it seems plausible that DNA damage by pathogens or herbivores can generate DNA fragments that trigger plant immunity *via* the same pathway that is activated by exogenously applied sonicated DNA. Similarly, the overall grade of conservation that characterises the DDR can serve as an argument that the assumed MRN-dependent activation of ATM (that is known from vertebrates but not yet form plants) is not too speculative. Nevertheless, the self/nonself-specific induction remains without an explanation. Although two transcriptomic studies revealed that exogenously applied fragments of self- and nonself-DNA induced expression of JA-biosynthetic and responsive genes in *A. thaliana* (24, 35), we observed that both mutants exhibited the self/nonself-DNA specific induction of JA that showed no detectable differences from the WT. We conclude that intact DDR machinery is required to activate the SA-dependent plant immunity response to extracellular DNA whereas an as-yet unknown mechanism controls the species-specific perception of DNA fragments as DAMPs that activate the JA-dependent wound response. Future studies will have to investigate whether the recently reported different mechanisms by which cGAS molecules from different mammalian species recognise their respective self-DNA (46) provide a starting point to identify the mechanisms that allow for a differential recognition of DNA in a single plant species.

## Data availability statement

The original contributions presented in the study are included in the article/**Supplementary Materials**, further inquiries can be directed to the corresponding author.

## Author contributions

IV-M, AH-E, and MH conceived and designed the experiments, IV-M performed the experiments and evaluated the data, IV-M and OM performed the statistical analysis, MH and IV-M wrote the first draft of the manuscript. All authors contributed to the article and approved the submitted version.
