# Supplementary material for: ATM and ATR, two central players of the DNA damage response, are involved in the induction of systemic acquired resistance by extracellular DNA, but not the plant wound response
Source: Front Immunol. 2023 May 15;14:1175786. doi: 10.3389/fimmu.2023.1175786 (PMC10225592; doi:10.3389/fimmu.2023.1175786)
Supplement: Supplementary file 1 [file DataSheet_1.docx]

***Supplementary Datasheet 1***

**corresponding to**

**ATM and ATR, two central players of the DNA damage response, are involved in the induction of systemic acquired resistance by extracellular DNA, but not the plant wound response**

**Isaac Vega-Muñoz, Octavio Martínez-de la Vega, Alfredo Herrera-Estrella, Martin Heil**

*** Correspondence:** Martin Heil: [martin.heil@cinvestav.mx](mailto:martin.heil@cinvestav.mx)

**Results of all statistical analysis are presented, titles of tables refer to the corresponding figure or panel.**

**Figure 1 | The Arabidopsis thaliana ROS response to exogenous DNA.**

**(1A)** Two-Way ANOVA of the effects of ‘time’ and self-DNA concentration (‘conc’) on the level of ROS in [mmol H_2_O_2_ g^-1^ leaf fresh weight]

|  | **Df** | **Sum Sq.** | **Mean Sq.** | **F value** | **Pr(>F)** | **Signif. Code** |
| --- | --- | --- | --- | --- | --- | --- |
| **Concentration** | 2 | 1041.0 | 520.5 | 23.448 | 1.45E-07 | *** |
| **Time** | 6 | 1081.5 | 180.2 | 8.120 | 7.74E-06 | *** |
| **Concentration x time** | 12 | 879.4 | 73.3 | 3.301 | 0.002 | ** |
| **Residuals** | 42 | 932.3 | 22.2 |  |  |  |

**(1B)** Log-logistic Effective Dose 50% for ROS response to self-DNA

Model fitted: Log-logistic (ED50 as parameter) (4 parms)

Parameter estimates:

|  | **Estimate** | **Std. Error** | **t-value** | **p-value** | **Signif. Code** |
| --- | --- | --- | --- | --- | --- |
| **Slope:(Intercept)** | -2.37646 | 2.72017 | -0.8736 | 0.39031 |  |
| **Lower Limit:(Intercept)** | 28.17456 | 1.38571 | 20.3322 | < 2e-16 | *** |
| **Upper Limit:(Intercept** | 78.06846 | 2.36270 | 33.0420 | < 2e-16 | *** |
| **ED50:(Intercept)** | 0.74604 | 0.35332 | 2.1115 | 0.04449 | * |

Residual standard error:

5.285247 (26 degrees of freedom

**(1B) Effective doses 5, 10, 50, 99%**

|  | **Estimate** | **Std. Error** | **Lower** | **Upper** |
| --- | --- | --- | --- | --- |
| **e:1:5** | 0.216108 | 0.2083 | -0.21206 | 0.644276 |
| **e:1:10** | 0.295953 | 0.179856 | -0.07375 | 0.665653 |
| **e:1:50** | 0.746041 | 0.353319 | 0.019783 | 1.472298 |
| **e:1:99** | 5.158376 | 13.80456 | -23.2173 | 33.53405 |

**Figure 2 | Self-nonself-specific induction of H_2_O_2_ and defence hormones by exogenous DNA**.

**(2A) One-Way ANOVA ROS 15 minutes self vs non self-DNA:**

|  | **Df** | **Sum Sq** | **Mean Sq** | **F value** | **Pr(>F)** | **Signif. Code** |
| --- | --- | --- | --- | --- | --- | --- |
| **Treatment** | 3 | 2847.757 | 949.2525 | 57.41974 | 8.74E-09 | *** |
| **Residuals** | 16 | 264.509 | 16.53181 | NA | NA |  |

**(2B) One-Way ANOVA JA 30 minutes self vs non self-DNA:**

|  | **Df** | **Sum Sq** | **Mean Sq** | **F value** | **Pr(>F)** | **Signif. Code** |
| --- | --- | --- | --- | --- | --- | --- |
| **Treatment** | 3 | 392.2802 | 130.7601 | 128.7833 | 2.05E-11 | *** |
| **Residuals** | 16 | 16.2456 | 1.01535 | NA | NA |  |

**(2C) One-Way ANOVA SA 24 hours self vs non self-DNA:**

|  | **Df** | **Sum Sq** | **Mean Sq** | **F value** | **Pr(>F)** | **Signif. Code** |
| --- | --- | --- | --- | --- | --- | --- |
| **Treatment** | 3 | 113.5131 | 37.8377 | 14.05362 | 9.52E-05 | *** |
| **Residuals** | 16 | 43.07809 | 2.692381 | NA | NA |  |

**Figure 3 | *In situ* damage by virulent bacteria or DNA-damaging molecules generates fragments with ROS-inducing activity**

**(3A) One-Way ANOVA eluted DNA:**

|  | **Df** | **Sum Sq** | **Mean Sq** | **F value** | **Pr(>F)** | **Signif. Code** |
| --- | --- | --- | --- | --- | --- | --- |
| **Treatment** | 7 | 0.012816 | 0.001831 | 0.367768 | 0.908 |  |
| **Residuals** | 16 | 0.079653 | 0.004978 | NA | NA |  |

**(3B) One-Way ANOVA ROS response to eluted DNA:**

|  | **Df** | **Sum Sq** | **Mean Sq** | **F value** | **Pr(>F)** | **Signif. Code** |
| --- | --- | --- | --- | --- | --- | --- |
| **Treatment** | 8 | 2532.754 | 316.5943 | 49.96631 | < 1e-16 | *** |
| **Residuals** | 36 | 228.1016 | 6.336154 | NA | NA |  |

**(3B) Results of post-hoc Tukey analyses (pairwise comparisons)**

|  | **Difference** | **Lwr** | **Upr** | **P** |
| --- | --- | --- | --- | --- |
| **Sonicated - Control** | 19.8340 | 14.5851 | 25.0830 | 0.0000 |
| **Control - Psg (-)** | 0.7666 | -4.4823 | 6.0156 | 0.9999 |
| **Control - Psg (+)** | 13.4988 | 8.2499 | 18.7478 | 0.0000 |
| **Control - Pst DC3000** | 18.7664 | 13.5174 | 24.0154 | 0.0000 |
| **Control - Bleomycin** | 8.1882 | 2.9393 | 13.4372 | 0.0003 |
| **Control - SA** | 12.9985 | 7.7495 | 18.2475 | 0.0000 |
| **Control - H_2_O_2_** | 2.7875 | -2.4615 | 8.0364 | 0.7121 |
| **Control - MSC** | 0.5769 | -4.6720 | 5.8259 | 1.0000 |
| **Sonicated - Psg (-)** | -19.0674 | -24.3164 | -13.8184 | 0.0000 |
| **Sonicated - Psg (+)** | -6.3352 | -11.5842 | -1.0863 | 0.0086 |
| **Somicated - Pst DC3000** | -1.0676 | -6.3166 | 4.1813 | 0.9989 |
| **Sonicated - Bleomycin** | -11.6458 | -16.8948 | -6.3968 | 0.0000 |
| **Sonicated - SA** | -6.8355 | -12.0845 | -1.5866 | 0.0036 |
| **Somicated - H_2_O_2_** | -17.0466 | -22.2955 | -11.7976 | 0.0000 |
| **Sonicated - MSC** | -19.2571 | -24.5061 | -14.0081 | 0.0000 |
| **Psg (-) - Psg (+)** | 12.7322 | 7.4832 | 17.9811 | 0.0000 |
| **Psg (-) - Pst DC3000** | 17.9998 | 12.7508 | 23.2487 | 0.0000 |
| **Psg (-) - Bleomycin** | 7.4216 | 2.1726 | 12.6706 | 0.0013 |
| **Psg (-) - SA** | 12.2319 | 6.9829 | 17.4808 | 0.0000 |
| **Psg (-) - H_2_O_2_** | 2.0208 | -3.2281 | 7.2698 | 0.9338 |
| **Psg (-) - MSC** | -0.1897 | -5.4387 | 5.0592 | 1.0000 |
| **Psg (+) - Pst DC3000** | 5.2676 | 0.0186 | 10.5165 | 0.0486 |
| **Psg (+) - Bleomycin** | -5.3106 | -10.5595 | -0.0616 | 0.0456 |
| **Psg (+) - SA** | -0.5003 | -5.7493 | 4.7487 | 1.0000 |
| **Psg (+) - H_2_O_2_** | -10.7113 | -15.9603 | -5.4624 | 0.0000 |
| **Psg (+) - MSC** | -12.9219 | -18.1709 | -7.6729 | 0.0000 |
| **Pst DC3000 - Bleomycin** | -10.5782 | -15.8271 | -5.3292 | 0.0000 |
| **Pst DC3000 - SA** | -5.7679 | -11.0169 | -0.5189 | 0.0222 |
| **Pst DC3000 - H_2_O_2_** | -15.9789 | -21.2279 | -10.7300 | 0.0000 |
| **Pst DC3000 - MSC** | -18.1895 | -23.4384 | -12.9405 | 0.0000 |
| **Bleomycin - SA** | 4.8103 | -0.4387 | 10.0592 | 0.0943 |
| **Bleomycin - H_2_O_2_** | -5.4008 | -10.6497 | -0.1518 | 0.0397 |
| **Bleomycin - MSC** | -7.6113 | -12.8603 | -2.3624 | 0.0009 |
| **SA - H_2_O_2_** | -10.2110 | -15.4600 | -4.9621 | 0.0000 |
| **SA - MSC** | -12.4216 | -17.6706 | -7.1726 | 0.0000 |
| **H_2_O_2_ - MSC** | -2.2106 | -7.4595 | 3.0384 | 0.8948 |

**P values are presented as a heatmap with light blue for 0, white for 0.05 and red for 1.0**

| **0** | **0.05** | **1** |
| --- | --- | --- |

**Figure 4 | Arabidopsis DDR mutant lines *atm* and *atr* are affected in self-nonself-specific induction of H_2_O_2_ and SA but exhibit normal JA**.

(**4A) Two-Way ANOVA ROS self vs self-DNA in DDR mutants:**

|  | **Df** | **Sum Sq.** | **Mean Sq.** | **F value** | **Pr(>F)** | **Signif. code** |
| --- | --- | --- | --- | --- | --- | --- |
| **Treatment** | 3 | 4977.176 | 1659.059 | 19.57743 | 1.98E-08 | *** |
| **Genotype** | 2 | 5957.919 | 2978.96 | 35.15269 | 3.96E-10 | *** |
| **Treatment x Genotype** | 6 | 4361.428 | 726.9047 | 8.577712 | 2.38E-06 | *** |
| **Residuals** | 48 | 4067.685 | 84.74343 | NA | NA |  |

**(4A) One-Way ANOVA ROS self vs self-DNA in wt:**

|  | **Df** | **Sum Sq** | **Mean Sq** | **F value** | **Pr(>F)** | **Signif. code** |
| --- | --- | --- | --- | --- | --- | --- |
| **Treatment** | 3 | 8404.497 | 2801.499 | 45.39741 | 4.74E-08 | *** |
| **Residuals** | 16 | 987.3687 | 61.71054 | NA | NA |  |

**(4A) One-Way ANOVA ROS self vs self-DNA in *atm*:**

|  | **Df** | **Sum Sq** | **Mean Sq** | **F value** | **Pr(>F)** | **Signif. code** |
| --- | --- | --- | --- | --- | --- | --- |
| **Treatment** | 3 | 833.6001 | 277.8667 | 2.615269 | 8.69E-02 | ** |
| **Residuals** | 16 | 1699.966 | 106.2478 | NA | NA |  |

**(4A) One-Way ANOVA ROS self vs self-DNA in atr:**

|  | **Df** | **Sum Sq** | **Mean Sq** | **F value** | **Pr(>F)** | **Signif. code** |
| --- | --- | --- | --- | --- | --- | --- |
| **Treatment** | 3 | 100.5077 | 33.50257 | 0.388337 | 7.63E-01 |  |
| **Residuals** | 16 | 1380.351 | 86.27191 | NA | NA |  |

**(4B) Two-Way ANOVA JA self vs self-DNA in DDR mutants:**

|  | **Df** | **Sum Sq.** | **Mean Sq.** | **F value** | **Pr(>F)** | **Signif. code** |
| --- | --- | --- | --- | --- | --- | --- |
| **Treatment** | 3 | 738.0978 | 246.0326 | 30.69857 | 3.15E-11 | *** |
| **Genotype** | 2 | 76.9346 | 38.4673 | 4.799733 | 1.26E-02 | * |
| **Treatment x Genotype** | 6 | 46.81241 | 7.802068 | 0.973498 | 4.53E-01 |  |
| **Residuals** | 48 | 384.6943 | 8.014465 | NA | NA |  |

**(4B) One-Way ANOVA JA self vs self-DNA in wt:**

|  | **Df** | **Sum Sq** | **Mean Sq** | **F value** | **Pr(>F)** | **Signif. Code** |
| --- | --- | --- | --- | --- | --- | --- |
| **Treatment** | 3 | 388.9172 | 129.6391 | 12.7341 | 1.65E-04 | *** |
| **Residuals** | 16 | 162.8874 | 10.18046 | NA | NA |  |

**(4B) One-Way ANOVA JA self vs self-DNA in atm:**

|  | **Df** | **Sum Sq** | **Mean Sq** | **F value** | **Pr(>F)** | **Signif. Code** |
| --- | --- | --- | --- | --- | --- | --- |
| **Treatment** | 3 | 199.4229 | 66.47431 | 9.247179 | 8.79E-04 | *** |
| **Residuals** | 16 | 115.0177 | 7.188604 | NA | NA |  |

**(4B) One-Way ANOVA JA self vs self-DNA in atr:**

|  | **Df** | **Sum Sq** | **Mean Sq** | **F value** | **Pr(>F)** | **Signif. code** |
| --- | --- | --- | --- | --- | --- | --- |
| **Treatment** | 3 | 196.5701 | 65.52336 | 9.817222 | 6.52E-04 | *** |
| **Residuals** | 16 | 106.7892 | 6.674327 | NA | NA |  |

**(4C) Two-Way ANOVA SA self vs self-DNA in DDR mutants:**

|  | **Df** | **Sum Sq.** | **Mean Sq.** | **F value** | **Pr(>F)** | **Signif. code** |
| --- | --- | --- | --- | --- | --- | --- |
| **Treatment** | 3 | 58.70599 | 19.56866 | 4.500779 | 7.32E-03 | ** |
| **Genotype** | 2 | 1.568749 | 0.784375 | 0.180406 | 8.35E-01 |  |
| **Treatment x Genotype** | 6 | 60.1988 | 10.03313 | 2.307614 | 4.89E-02 | * |
| **Residuals** | 48 | 208.6962 | 4.347839 | NA | NA |  |

**(4C) One-Way ANOVA SA self vs self-DNA in wt:**

|  | **Df** | **Sum Sq** | **Mean Sq** | **F value** | **Pr(>F)** | **Signif. Code** |
| --- | --- | --- | --- | --- | --- | --- |
| **Treatment** | 3 | 113.3598 | 37.7866 | 9.75182 | 6.75E-04 | *** |
| **Residuals** | 16 | 61.99721 | 3.874825 | NA | NA |  |

**(4C) One-Way ANOVA SA self vs self-DNA in atm:**

|  | **Df** | **Sum Sq** | **Mean Sq** | **F value** | **Pr(>F)** | **Signif. Code** |
| --- | --- | --- | --- | --- | --- | --- |
| **Treatment** | 3 | 1.814954 | 0.604985 | 0.12376 | 9.45E-01 |  |
| **Residuals** | 16 | 78.21381 | 4.888363 | NA | NA |  |

**(4C) One-Way ANOVA SA self vs self-DNA in atr:**

|  | **Df** | **Sum Sq** | **Mean Sq** | **F value** | **Pr(>F)** | **Signif. Code** |
| --- | --- | --- | --- | --- | --- | --- |
| **Treatment** | 3 | 3.730034 | 1.243345 | 0.290479 | 8.32E-01 |  |
| **Residuals** | 16 | 68.48523 | 4.280327 | NA | NA |  |

**Results of post-hoc Tukey analyses (pairwise comparisons) for the effects of DNA treatment on SA levels in wt, *atm* and *atr***

| WT |  |
| --- | --- |
| col:wt-ctl:wt | 0.0203 |
| cvi:wt-ctl:wt | 0.1041 |
| br:wt-ctl:wt | 1.0000 |
| cvi:wt-col:wt | 1.0000 |
| br:wt-col:wt | 0.0098 |
| br:wt-cvi:wt | 0.0564 |
|  |  |
| ATM |  |
| col:*atm*-ctl:*atm* | 1.0000 |
| cvi:*atm*-ctl:*atm* | 1.0000 |
| br:*atm*-ctl:*atm* | 1.0000 |
| cvi:*atm*-col:*atm* | 1.0000 |
| br:*atm*-col:*atm* | 1.0000 |
| br:*atm*-cvi:*atm* | 1.0000 |
|  |  |
| ATR |  |
| col:*atr*-ctl:*atr* | 1.0000 |
| cvi:*atr*-ctl:*atr* | 1.0000 |
| br:*atr*-ctl:*atr* | 1.0000 |
| cvi:*atr*-col:*atr* | 1.0000 |
| br:*atr*-col:*atr* | 0.9992 |
| br:*atr*-cvi:*atr* | 0.9999 |
|  |  |
| ATM-WT |  |
| ctl:*atm*-ctl:wt | 0.9953 |
| ctl:*atm*-col:wt | 0.2497 |
| ctl:*atm*-cvi:wt | 0.6386 |
| ctl:*atm*-br:wt | 0.9742 |
| col:*atm*-col:wt | 0.5738 |
| cvi:*atm*-cvi:wt | 0.9320 |
| br:*atm*-br:wt | 0.8826 |
| col:*atm*-ctl:wt | 0.9031 |
| cvi:*atm*-ctl:wt | 0.8900 |
| br:*atm*-ctl:wt | 0.9594 |
| col:*atm*-cvi:wt | 0.9215 |
| cvi:*atm*-col:wt | 0.5972 |
| br:*atm*-col:wt | 0.4405 |
| br:*atm*-cvi:wt | 0.8405 |
| col:*atm*-br:wt | 0.7841 |
| cvi:*atm*-br:wt | 0.7642 |
|  |  |
| ATR WT |  |
| ctl:*atr*-ctl:wt | 0.8383 |
| ctl:*atr*-col:wt | 0.6750 |
| ctl:*atr*-cvi:wt | 0.9603 |
| ctl:*atr*-br:wt | 0.6922 |
| col:*atr*-col:wt | 0.8279 |
| cvi:*atr*-cvi:wt | 0.9793 |
| br:*atr*-br:wt | 0.9582 |
| col:*atr*-ctl:wt | 0.6885 |
| cvi:*atr*-ctl:wt | 0.7745 |
| br:*atr*-ctl:wt | 0.9907 |
| col:*atr*-cvi:wt | 0.9917 |
| cvi:*atr*-col:wt | 0.7500 |
| br:*atr*-col:wt | 0.2952 |
| br:*atr*-cvi:wt | 0.6988 |
| col:*atr*-br:wt | 0.5188 |
| cvi:*atr*-br:wt | 0.6134 |
|  |  |
| ATM ATR |  |
| cvi:*atr*-ctl:*atm* | 0.9995 |
| ctl:*atr*-ctl:*atm* | 0.9999 |
| col:*atr*-br:*atm* | 1.0000 |
| br:*atr*-cvi:*atm* | 1.0000 |
| cvi:*atr*-br:*atm* | 1.0000 |
| br:*atr*-col:*atm* | 1.0000 |
| col:*atr*-col:*atm* | 1.0000 |
| col:*atr*-cvi:*atm* | 1.0000 |
| ctl:*atr*-br:*atm* | 1.0000 |
| cvi:*atr*-col:*atm* | 1.0000 |
| br:*atr*-br:*atm* | 1.0000 |
| cvi:*atr*-cvi:*atm* | 1.0000 |
| ctl:*atr*-col:*atm* | 1.0000 |
| br:*atr*-ctl:*atm* | 1.0000 |
| ctl:*atr*-cvi:*atm* | 1.0000 |

**P values are presented as a heatmap with light blue for 0, pink for 0.05 and red for 1.0**

| **0** | **0.05** | **1** |
| --- | --- | --- |

**Figure 5 | Arabidopsis DDR mutant line *atm* but not *atr* is affected in the immunity to a bacterial pathogen.**

**(5B) Two-Way ANOVA Pathogen infection self vs self-DNA in DDR mutants:**

|  | **Df** | **Sum Sq.** | **Mean Sq.** | **F value** | **Pr(>F)** | **Signif. code** |
| --- | --- | --- | --- | --- | --- | --- |
| **Treatment** | 3 | 1.06E+10 | 3.55E+09 | 9.372655 | 5.51E-05 | *** |
| **Genotype** | 2 | 3.15E+09 | 1.57E+09 | 4.159834 | 2.16E-02 | * |
| **Treatment x Genotype** | 6 | 9.43E+09 | 1.57E+09 | 4.15387 | 1.94E-03 | ** |
| **Residuals** | 48 | 1.82E+10 | 3.78E+08 | NA | NA |  |

**(5B) One-Way ANOVA Pathogen infection self vs self-DNA in wt:**

|  | **Df** | **Sum Sq** | **Mean Sq** | **F value** | **Pr(>F)** | **Signif. Code** |
| --- | --- | --- | --- | --- | --- | --- |
| **Treatment** | 3 | 6.47E+09 | 2.16E+09 | 11.23935 | 3.24E-04 | *** |
| **Residuals** | 16 | 3.07E+09 | 1.92E+08 | NA | NA |  |

**(5B) One-Way ANOVA Pathogen infection self vs self-DNA in atm:**

|  | **Df** | **Sum Sq** | **Mean Sq** | **F value** | **Pr(>F)** | **Signif. Code** |
| --- | --- | --- | --- | --- | --- | --- |
| **Treatment** | 3 | 1.76E+09 | 5.88E+08 | 1.241785 | 3.27E-01 |  |
| **Residuals** | 16 | 7.57E+09 | 4.73E+08 | NA | NA |  |

**(5B) One-Way ANOVA Pathogen infection self vs self-DNA in atr:**

|  | **Df** | **Sum Sq** | **Mean Sq** | **F value** | **Pr(>F)** | **Signif. Code** |
| --- | --- | --- | --- | --- | --- | --- |
| **Treatment** | 3 | 1.18E+10 | 3.94E+09 | 8.397764 | 1.40E-03 | ** |
| **Residuals** | 16 | 7.51E+09 | 4.69E+08 | NA | NA |  |

**Figure 6 | Presence and methylation of CpG motifs have minor effects on ROS-inducing properties of DNA**.

**(6A) Two-Way ANOVA ROS response to different DNA methylation status:**

|  | **Df** | **Sum Sq.** | **Mean Sq.** | **F value** | **Pr(>F)** | **Signif. code** |
| --- | --- | --- | --- | --- | --- | --- |
| **Treatment** | 2 | 308.0664 | 154.0332 | 2.826894 | 6.91E-02 |  |
| **Genotype** | 3 | 4503.489 | 1501.163 | 27.55009 | 1.65E-10 | *** |
| **Treatment x Genotype** | 6 | 466.1394 | 77.68989 | 1.425804 | 2.24E-01 |  |
| **Residuals** | 48 | 2615.448 | 54.4885 | NA | NA |  |

**(6A) One-Way ANOVA ROS response to natural DNA:**

|  | **Df** | **Sum Sq** | **Mean Sq** | **F value** | **Pr(>F)** | **Signif. Code** |
| --- | --- | --- | --- | --- | --- | --- |
| **Treatment** | 3 | 2602.91 | 867.6366 | 12.76953 | 1.63E-04 | *** |
| **Residuals** | 16 | 1087.133 | 67.94583 | NA | NA |  |

**(6A) One-Way ANOVA ROS response to M.SssI-methylated DNA:**

|  | **Df** | **Sum Sq** | **Mean Sq** | **F value** | **Pr(>F)** | **Signif. Code** |
| --- | --- | --- | --- | --- | --- | --- |
| **Treatment** | 3 | 1064.196 | 354.7318 | 6.932224 | 3.34E-03 | ** |
| **Residuals** | 16 | 818.743 | 51.17144 | NA | NA |  |

**(6A) One-Way ANOVA ROS response to PCR-generated DNA:**

|  | **Df** | **Sum Sq** | **Mean Sq** | **F value** | **Pr(>F)** | **Signif. Code** |
| --- | --- | --- | --- | --- | --- | --- |
| **Treatment** | 3 | 1302.523 | 434.1744 | 9.790119 | 6.61E-04 | *** |
| **Residuals** | 16 | 709.5716 | 44.34823 | NA | NA |  |

**(6B) One-way ANOVA ROS response to immunostimulatory oODNs:**

|  | **Df** | **Sum Sq** | **Mean Sq** | **F value** | **Pr(>F)** | **Signif. Code** |
| --- | --- | --- | --- | --- | --- | --- |
| **Treatment** | 6 | 1228.343 | 204.7238 | 6.096028 | 3.54E-04 | *** |
| **Residuals** | 28 | 940.328 | 33.58314 | NA | NA |  |

**Results of post-hoc Tukey analyses (pairwise comparisons) for the effects of treatment with different ODNs**

|  | **Difference** | **Lwr** | **Upr** | **P** |
| --- | --- | --- | --- | --- |
| **Control - 2006s** | 16.8875 | 5.2612 | 28.5138 | 0.0014 |
| **Control - 2006c** | 18.1553 | 6.5290 | 29.7816 | 0.0006 |
| **Control - 2006ds** | 17.7292 | 6.1029 | 29.3555 | 0.0008 |
| **Control - IMT504s** | 14.5292 | 2.9029 | 26.1555 | 0.0075 |
| **Control - IMT504c** | 13.6114 | 1.9851 | 25.2377 | 0.0139 |
| **Control - IMT504ds** | 17.1186 | 5.4923 | 28.7449 | 0.0012 |
|  |  |  |  |  |
| **2006s - 2006c** | 1.2678 | -10.3585 | 12.8941 | 0.9998 |
| **2006s - 2006ds** | 0.8417 | -10.7846 | 12.4680 | 1.0000 |
| **2006s - IMT504s** | -2.3583 | -13.9846 | 9.2680 | 0.9946 |
| **2006s - IMT504c** | -3.2761 | -14.9024 | 8.3502 | 0.9705 |
| **2006s - IMT504ds** | 0.2311 | -11.3952 | 11.8574 | 1.0000 |
|  |  |  |  |  |
| **2006c - 2006ds** | -0.4261 | -12.0524 | 11.2002 | 1.0000 |
| **2006c - IMT504s** | -3.6261 | -15.2524 | 8.0002 | 0.9522 |
| **2006c - IMT504c** | -4.5439 | -16.1702 | 7.0824 | 0.8724 |
| **2006c - IMT504ds** | -1.0367 | -12.6630 | 10.5896 | 0.9999 |
|  |  |  |  |  |
| **2006ds - IMT504s** | -3.2000 | -14.8263 | 8.4263 | 0.9737 |
| **2006ds - IMT504c** | -4.1178 | -15.7441 | 7.5085 | 0.9154 |
| **2006ds - IMT504ds** | -0.6106 | -12.2369 | 11.0157 | 1.0000 |
|  |  |  |  |  |
| **IMT504s - IMT504c** | -0.9178 | -12.5441 | 10.7085 | 1.0000 |
| **IMT504s - IMT504ds** | 2.5894 | -9.0369 | 14.2157 | 0.9911 |
| **IMT504c - IMT504ds** | 3.5072 | -8.1191 | 15.1335 | 0.9591 |

**Supplementary Figure S2 | Self-nonself-specific time-curve induction of JA and SA by exogenous DNA**

**(2A) Self-nonself-specific time-curve induction of JA by exogenous DNA**

|  | **Df** | **Sum Sq.** | **Mean Sq.** | **F value** | **Pr(>F)** | **Signif. code** |
| --- | --- | --- | --- | --- | --- | --- |
| **Treatment** | 3 | 257.6712 | 85.89041 | 59.97316 | < 1e-16 | *** |
| **Time** | 4 | 767.5436 | 191.8859 | 133.9847 | < 1e-16 | *** |
| **Treatment x Time** | 12 | 309.7018 | 25.80849 | 18.02083 | < 1e-16 | *** |
| **Residuals** | 80 | 114.5718 | 1.432147 | NA | NA |  |

**(2B) Self-nonself-specific time-curve induction of SA by exogenous DNA**

|  | **Df** | **Sum Sq.** | **Mean Sq.** | **F value** | **Pr(>F)** | **Signif. code** |
| --- | --- | --- | --- | --- | --- | --- |
| **Treatment** | 3 | 152.0442 | 50.6814 | 27.59376 | 1.43E-11 | *** |
| **Time** | 3 | 56.9565 | 18.9855 | 10.33676 | 1.23E-05 | *** |
| **Treatment x Time** | 9 | 69.36474 | 7.707193 | 4.196222 | 2.71E-04 | *** |
| **Residuals** | 64 | 117.5487 | 1.836698 | NA | NA |  |
