# Supplementary material for: ATM and ATR, two central players of the DNA damage response, are involved in the induction of systemic acquired resistance by extracellular DNA, but not the plant wound response
Source: Front Immunol. 2023 May 15;14:1175786. doi: 10.3389/fimmu.2023.1175786 (PMC10225592; doi:10.3389/fimmu.2023.1175786)
Supplement: Supplementary file 2 [file Presentation_1.pptx]

## Slide 1
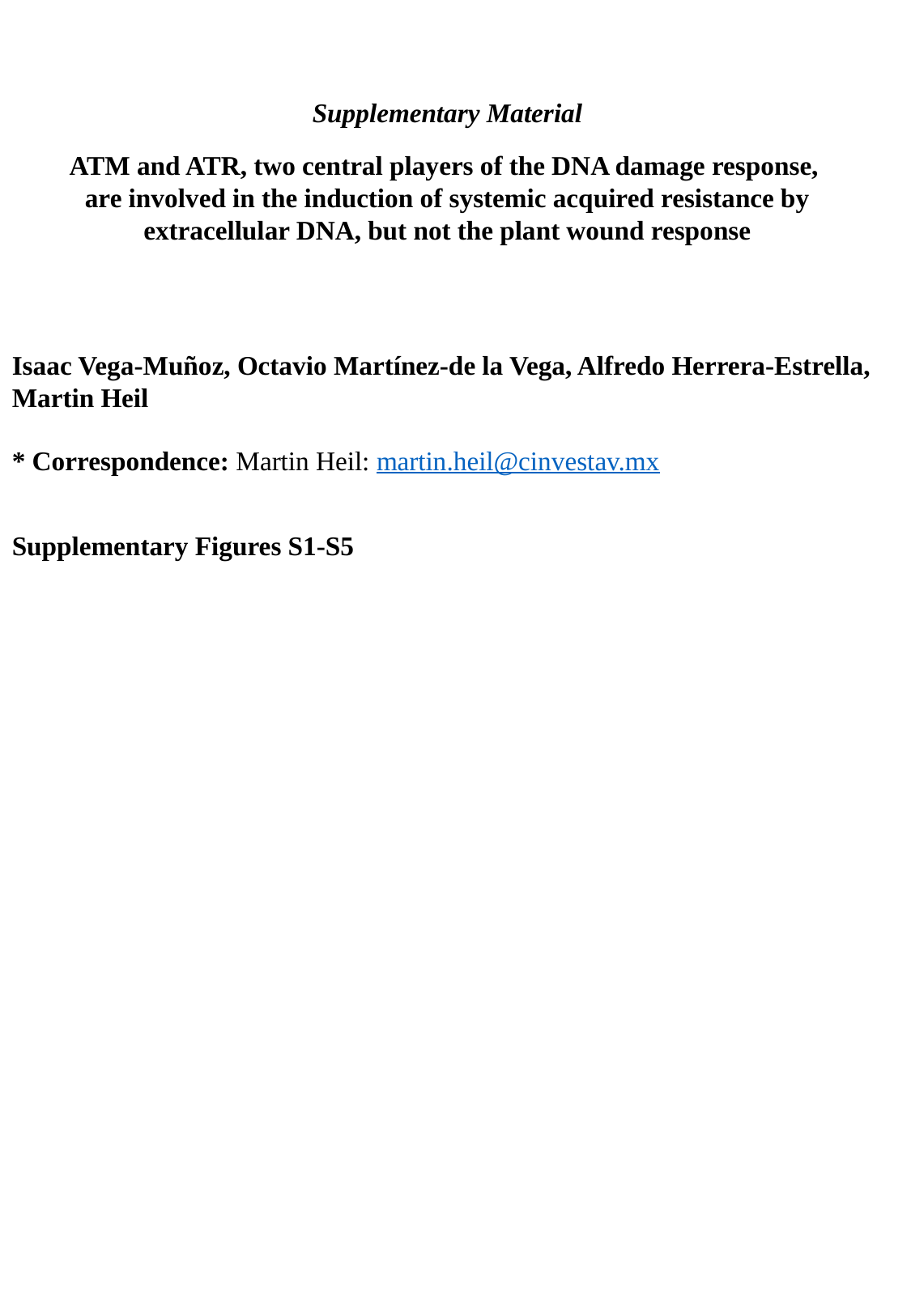

Supplementary Material
ATM and ATR, two central players of the DNA damage response,
are involved in the induction of systemic acquired resistance by extracellular DNA, but not the plant wound response
Isaac Vega-Muñoz, Octavio Martínez-de la Vega, Alfredo Herrera-Estrella, Martin Heil
* Correspondence: Martin Heil: martin.heil@cinvestav.mx
Supplementary Figures S1-S5

## Slide 2
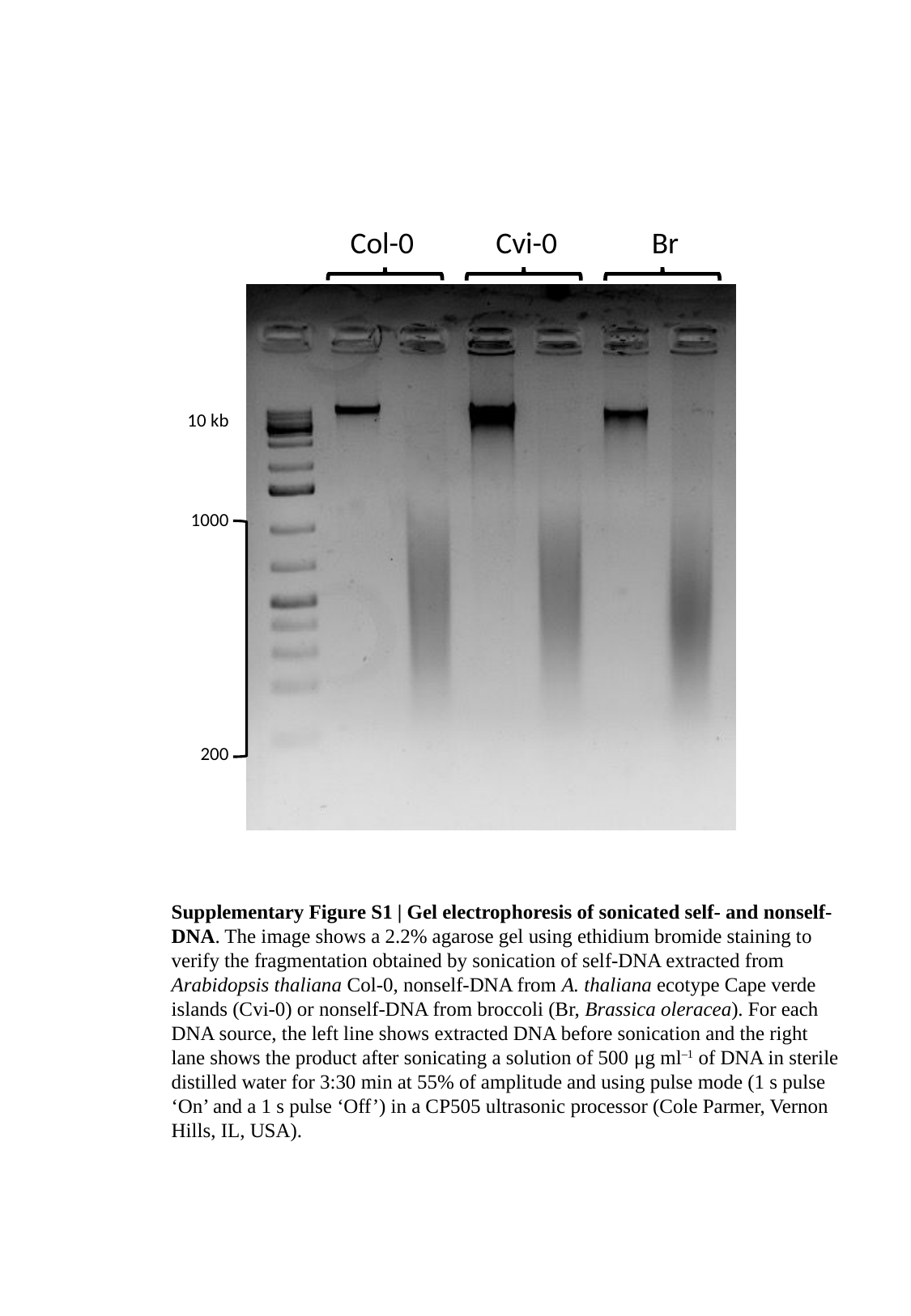

Col-0
Cvi-0
Br
10 kb
1000
200
Supplementary Figure S1 | Gel electrophoresis of sonicated self- and nonself-DNA. The image shows a 2.2% agarose gel using ethidium bromide staining to verify the fragmentation obtained by sonication of self-DNA extracted from Arabidopsis thaliana Col-0, nonself-DNA from A. thaliana ecotype Cape verde islands (Cvi-0) or nonself-DNA from broccoli (Br, Brassica oleracea). For each DNA source, the left line shows extracted DNA before sonication and the right lane shows the product after sonicating a solution of 500 μg ml–1 of DNA in sterile distilled water for 3:30 min at 55% of amplitude and using pulse mode (1 s pulse ‘On’ and a 1 s pulse ‘Off’) in a CP505 ultrasonic processor (Cole Parmer, Vernon Hills, IL, USA).

## Slide 3
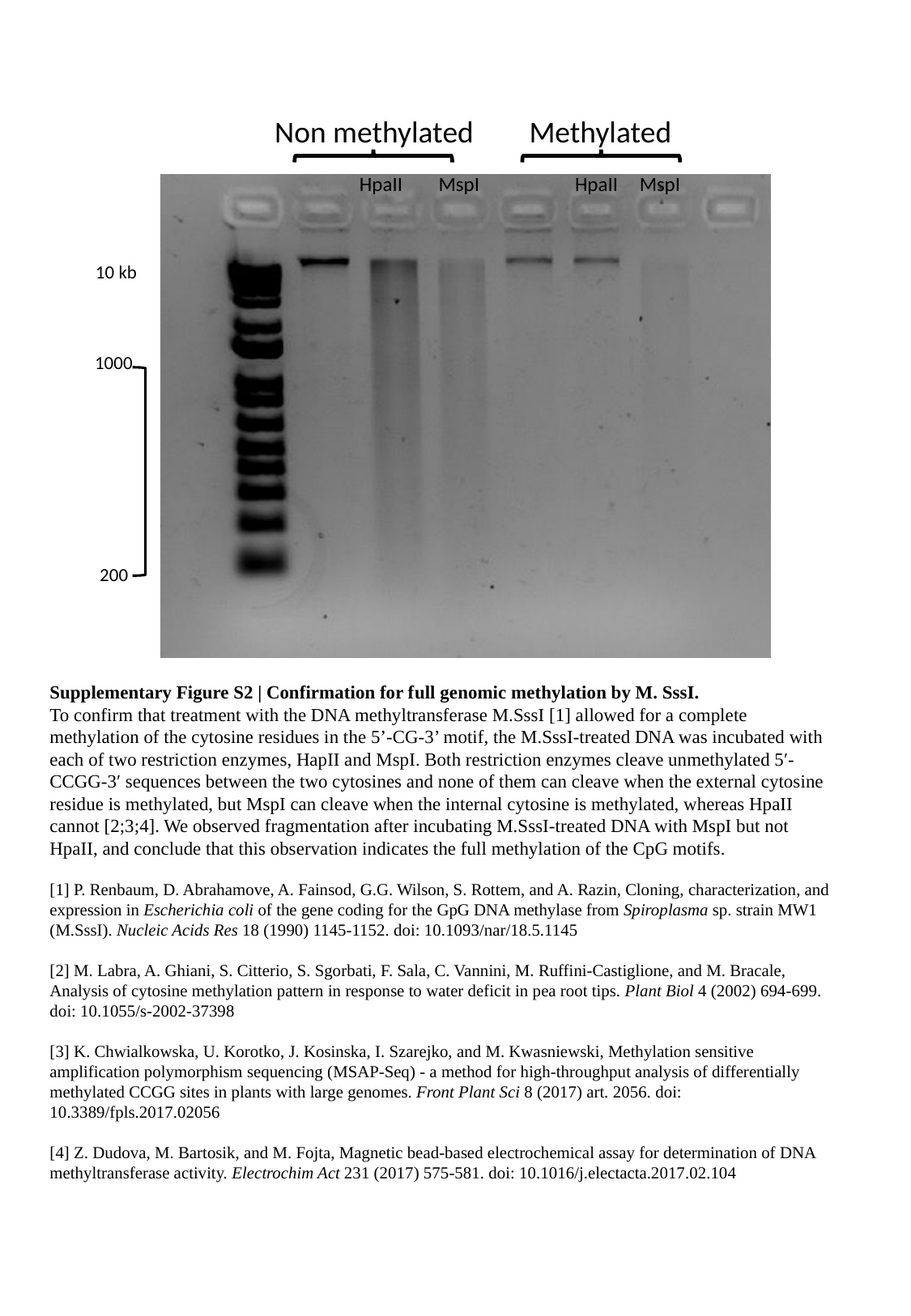

Non methylated
Methylated
HpaII
MspI
HpaII
MspI
10 kb
1000
200
Supplementary Figure S2 | Confirmation for full genomic methylation by M. SssI.
To confirm that treatment with the DNA methyltransferase M.SssI [1] allowed for a complete methylation of the cytosine residues in the 5’-CG-3’ motif, the M.SssI-treated DNA was incubated with each of two restriction enzymes, HapII and MspI. Both restriction enzymes cleave unmethylated 5′-CCGG-3′ sequences between the two cytosines and none of them can cleave when the external cytosine residue is methylated, but MspI can cleave when the internal cytosine is methylated, whereas HpaII cannot [2;3;4]. We observed fragmentation after incubating M.SssI-treated DNA with MspI but not HpaII, and conclude that this observation indicates the full methylation of the CpG motifs.
[1] P. Renbaum, D. Abrahamove, A. Fainsod, G.G. Wilson, S. Rottem, and A. Razin, Cloning, characterization, and expression in Escherichia coli of the gene coding for the GpG DNA methylase from Spiroplasma sp. strain MW1 (M.SssI). Nucleic Acids Res 18 (1990) 1145-1152. doi: 10.1093/nar/18.5.1145
[2] M. Labra, A. Ghiani, S. Citterio, S. Sgorbati, F. Sala, C. Vannini, M. Ruffini-Castiglione, and M. Bracale, Analysis of cytosine methylation pattern in response to water deficit in pea root tips. Plant Biol 4 (2002) 694-699. doi: 10.1055/s-2002-37398
[3] K. Chwialkowska, U. Korotko, J. Kosinska, I. Szarejko, and M. Kwasniewski, Methylation sensitive amplification polymorphism sequencing (MSAP-Seq) - a method for high-throughput analysis of differentially methylated CCGG sites in plants with large genomes. Front Plant Sci 8 (2017) art. 2056. doi: 10.3389/fpls.2017.02056
[4] Z. Dudova, M. Bartosik, and M. Fojta, Magnetic bead-based electrochemical assay for determination of DNA methyltransferase activity. Electrochim Act 231 (2017) 575-581. doi: 10.1016/j.electacta.2017.02.104

## Slide 4
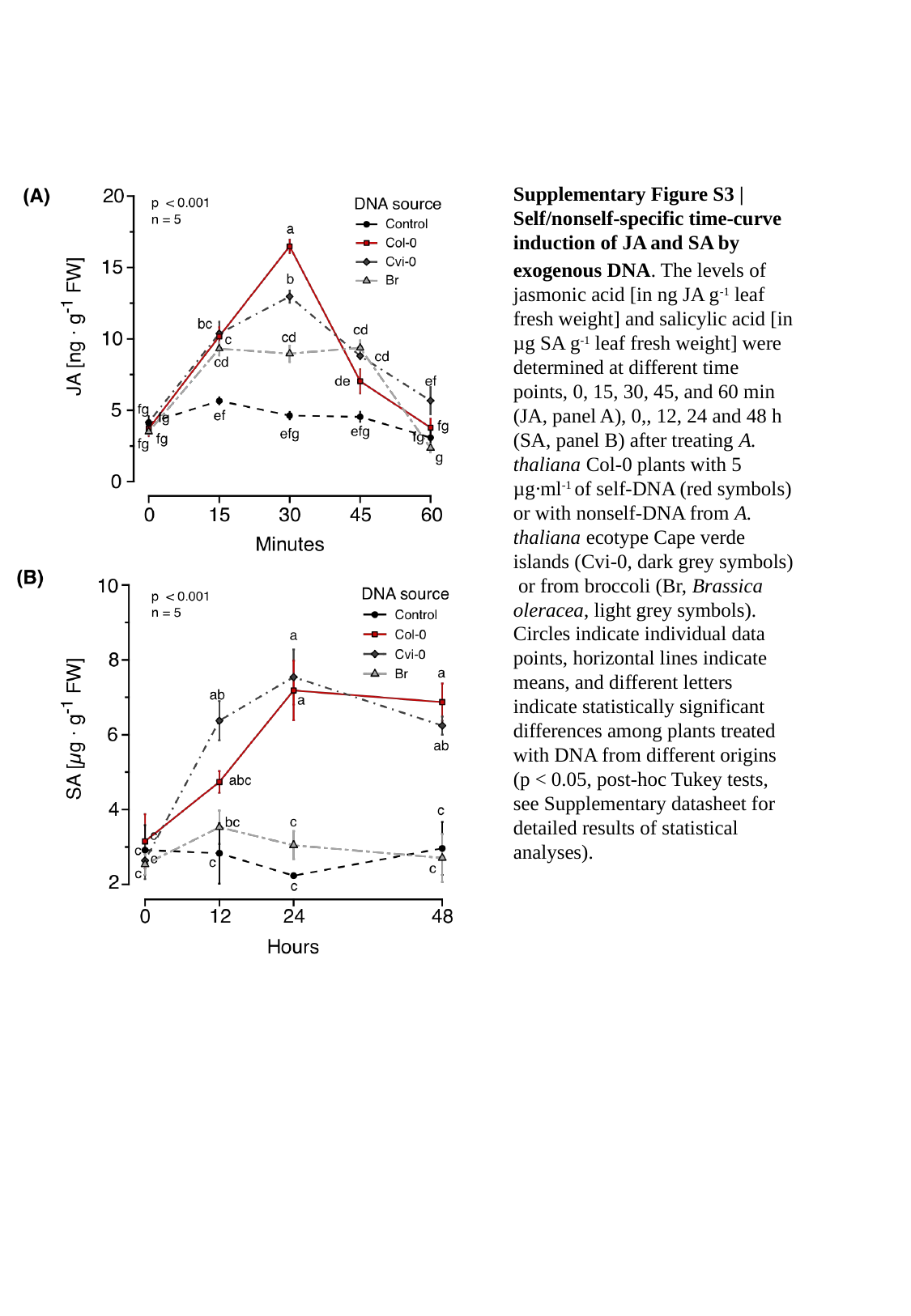

Supplementary Figure S3 | Self/nonself-specific time-curve induction of JA and SA by exogenous DNA. The levels of jasmonic acid [in ng JA g-1 leaf fresh weight] and salicylic acid [in µg SA g-1 leaf fresh weight] were determined at different time points, 0, 15, 30, 45, and 60 min (JA, panel A), 0,, 12, 24 and 48 h (SA, panel B) after treating A. thaliana Col-0 plants with 5 µg·ml-1 of self-DNA (red symbols) or with nonself-DNA from A. thaliana ecotype Cape verde islands (Cvi-0, dark grey symbols) or from broccoli (Br, Brassica oleracea, light grey symbols). Circles indicate individual data points, horizontal lines indicate means, and different letters indicate statistically significant differences among plants treated with DNA from different origins (p < 0.05, post-hoc Tukey tests, see Supplementary datasheet for detailed results of statistical analyses).

## Slide 5
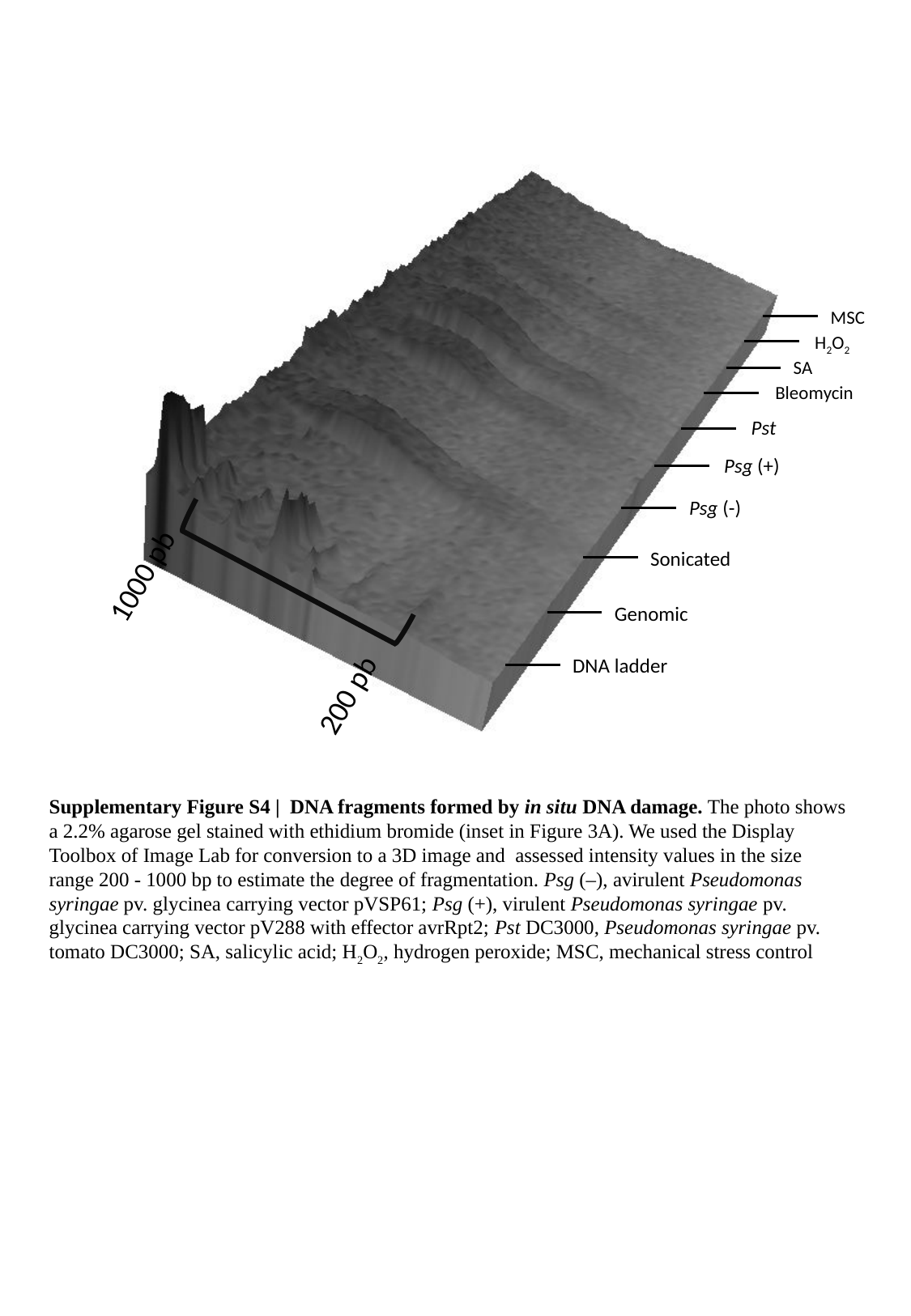

Fragments percentage below 1000 bp
MSC
H2O2
SA
Bleomycin
Pst
Psg (+)
Psg (-)
Sonicated
Genomic
DNA ladder
1000 pb
200 pb
Supplementary Figure S4 | DNA fragments formed by in situ DNA damage. The photo shows a 2.2% agarose gel stained with ethidium bromide (inset in Figure 3A). We used the Display Toolbox of Image Lab for conversion to a 3D image and assessed intensity values in the size range 200 - 1000 bp to estimate the degree of fragmentation. Psg (–), avirulent Pseudomonas syringae pv. glycinea carrying vector pVSP61; Psg (+), virulent Pseudomonas syringae pv. glycinea carrying vector pV288 with effector avrRpt2; Pst DC3000, Pseudomonas syringae pv. tomato DC3000; SA, salicylic acid; H2O2, hydrogen peroxide; MSC, mechanical stress control

## Slide 6
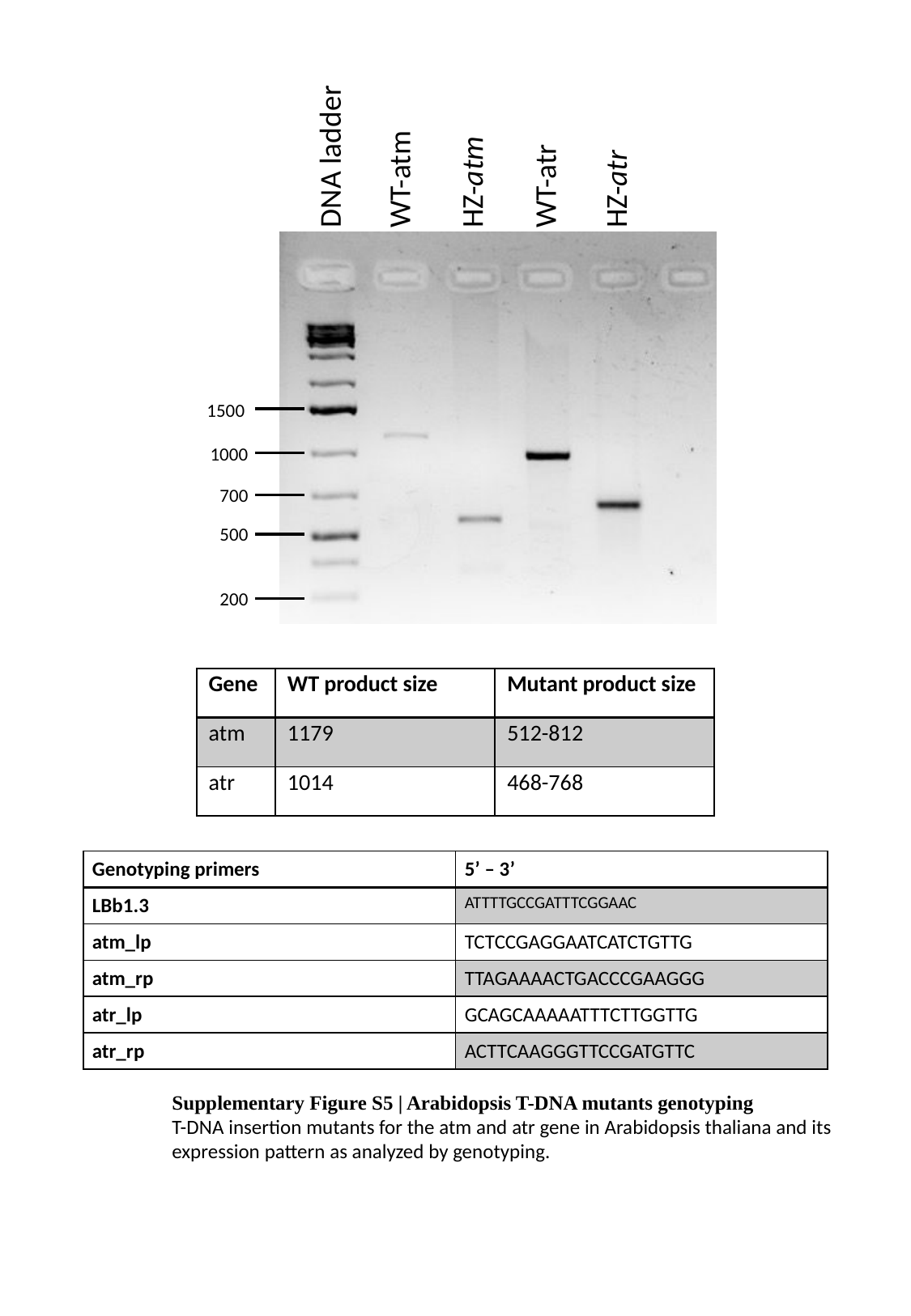

DNA ladder
WT-atm
HZ-atm
WT-atr
HZ-atr
1500
1000
700
500
200
| Gene | WT product size | Mutant product size |
| --- | --- | --- |
| atm | 1179 | 512-812 |
| atr | 1014 | 468-768 |
| Genotyping primers | 5’ – 3’ |
| --- | --- |
| LBb1.3 | ATTTTGCCGATTTCGGAAC |
| atm\_lp | TCTCCGAGGAATCATCTGTTG |
| atm\_rp | TTAGAAAACTGACCCGAAGGG |
| atr\_lp | GCAGCAAAAATTTCTTGGTTG |
| atr\_rp | ACTTCAAGGGTTCCGATGTTC |
Supplementary Figure S5 | Arabidopsis T-DNA mutants genotyping
T-DNA insertion mutants for the atm and atr gene in Arabidopsis thaliana and its expression pattern as analyzed by genotyping.
